# Attributable mortality due to nosocomial sepsis in Brazilian hospitals: a case–control study

**DOI:** 10.1186/s13613-023-01123-y

**Published:** 2023-04-26

**Authors:** Fernando G. Zampieri, Alexandre B. Cavalcanti, Leandro U. Taniguchi, Thiago C. Lisboa, Ary Serpa-Neto, Luciano C. P. Azevedo, Antonio Paulo Nassar, Tamiris A. Miranda, Samara P. C. Gomes, Meton S. de Alencar Filho, Rodrigo T. Amancio da Silva, Fabio Holanda Lacerda, Viviane Cordeiro Veiga, Airton Leonardo de Oliveira Manoel, Rodrigo S. Biondi, Israel S. Maia, Wilson J. Lovato, Claudio Dornas de Oliveira, Felipe Dal Pizzol, Milton Caldeira Filho, Cristina P. Amendola, Glauco A. Westphal, Rodrigo C. Figueiredo, Eliana B. Caser, Lanese M. de Figueiredo, Flávio Geraldo R. de Freitas, Sergio S. Fernandes, Andre Luiz N. Gobatto, Jorge Luiz R. Paranhos, Rodrigo Morel V. de Melo, Michelle T. Sousa, Guacyra Margarita B. de Almeida, Bianca R. Ferronatto, Denise M. Ferreira, Fernando J. S. Ramos, Marlus M. Thompson, Cintia M. C. Grion, Renato Hideo Nakagawa Santos, Lucas P. Damiani, Flavia R. Machado, for the MAPA investigators, the BRICNet, Barbara Macedo, Fabio S. Coutinho, Jussara A. Arraes, Viviane S. N. Xavier, Eliana V. N. Martins, Juliana Chaves Coelho, Silvana S. Santos, Andreia Pardini, Cassio Luis Zandonai, Julia B. de Carvalho, Isabela O. B. Louredo, Renata C. Gonçalves, Micheli C. Arruda, Mariana Regina da Cunha, Mariana Bonomini F. de Almeida, Juliano Ramos, Bruna M. Binda, Priscila L. S. Almeida, Marcia Maria R. de Oliveira, Luciana S. de Mattos, Samara G. da Silva, Daniela C. Dorta, Martha Hadrich, Fernanda A. F. Gonçalves, Kaytiussia R. de Sena, Pamella M. dos Prazeres, Josiane Festti

**Affiliations:** 1 HCor Research Institute, Rua Desembargador Eliseu Guilherme, 200, 8th Floor, São Paulo, Brazil; 2 Department of Critical Care Medicine, Faculty of Medicine and Dentistry, University of Alberta, 2-124E Clinical Sciences Building, 8440-112 St NW, Edmonton, AB T6G2B7 Canada; 3 Intensive Care Unit, Emergency Medicine Discipline, Hospital das Clínicas da Faculdade de Medicina da Universidade de São Paulo, São Paulo, Brazil; 4 Intensive Care Unit, Hospital Sírio-Libanês, São Paulo, SP Brazil; 5 Unidade de Terapia Intensiva, Santa Casa de Misericórdia de Porto Alegre, Porto Alegre, RS Brazil; 6 Department of Critical Care Medicine, Hospital Israelita Albert Einstein, Sao Paulo, Brazil; 7 Australian and New Zealand Intensive Care Research Centre (ANZIC-RC), School of Public Health and Preventive Medicine, Monash University, Melbourne, Australia; 8 Department of Intensive Care, Austin Hospital, Melbourne, Australia; 9 Intensive Care Unit, AC Camargo Cancer Center, São Paulo, SP Brazil; 10 Hospital Maternidade São Vicente de Paulo, Barbalha, CE Brazil; 11 Hospital Federal dos Servidores do Estado, Rio de Janeiro, RJ Brazil; 12 Hospital da Luz, São Paulo, SP Brazil; 13 BP-A Beneficência Portuguesa de São Paulo, Sao Paulo, SP Brazil; 14 Hospital Paulistano, São Paulo, SP Brazil; 15 Instituto de Cardiologia do Distrito Federal, Brasilia, DF Brazil; 16 Hospital Nereu Ramos, Florianópolis, SC Brazil; 17 Hospital Baía Sul, Florianópolis, SC Brazil; 18 Hospital das Clínicas da Faculdade de Medicina de Ribeirão Preto, Ribeirão Preto, SP Brazil; 19 Santa Casa de Misericórdia Belo Horizonte, Belo Horizonte, MG Brazil; 20 Hospital São José, Criciúma, SC Brazil; 21 Hospital Dona Helena, Joinville, SC Brazil; 22 Hospital de Amor-Fundação PIO XII, Barretos, SP Brazil; 23 Centro Hospitalar Unimed, Joinville, SC Brazil; 24 Hospital Maternidade São José, Colatina, ES Brazil; 25 Hospital Unimed Vitória, Vitória, ES Brazil; 26 Hospital Distrital Evandro Ayres de Moura Antônio Bezerra, Fortaleza, CE Brazil; 27 Hospital e Maternidade Sepaco, Sao Paulo, SP Brazil; 28 Hospital Japones Santa Cruz, Sao Paulo, SP Brazil; 29 Hospital da Cidade, Salvador, BA Brazil; 30 Santa Casa de Misericórdia de São João Del Rei, Belo Horizonte, MG Brazil; 31 Hospital Ana Nery, Salvador, BA Brazil; 32 Fundação São Francisco de Assis, Belo Horizonte, MG Brazil; 33 Hospital Regional Dr. Clodolfo Rodrigues de Melo, Maceio, AL Brazil; 34 Hospital Erasto Gaertner, Curitiba, PR Brazil; 35 Hospital das Clínicas da Universidade Federal de Goiás, Goiânia, GO Brazil; 36 Hospital Evangélico de Cachoeiro de Itapemirim, Cachoeiro de Itapemirim, ES Brazil; 37 Hospital Universitário Regional do Norte do Paraná, Londrina, PR Brazil; 38 Department of Anesthesiology, Pain and Critical Care-Hospital São Paulo, Escola Paulista de Medicina, Universidade Federal de Sao Paulo, Sao Paulo, SP Brazil

**Keywords:** Sepsis, Attributable mortality, Epidemiology

## Abstract

**Background:**

Nosocomial sepsis is a major healthcare issue, but there are few data on estimates of its attributable mortality. We aimed to estimate attributable mortality fraction (AF) due to nosocomial sepsis.

**Methods:**

Matched 1:1 case–control study in 37 hospitals in Brazil. Hospitalized patients in participating hospitals were included. Cases were hospital non-survivors and controls were hospital survivors, which were matched by admission type and date of discharge. Exposure was defined as occurrence of nosocomial sepsis, defined as antibiotic prescription plus presence of organ dysfunction attributed to sepsis without an alternative reason for organ failure; alternative definitions were explored. Main outcome measurement was nosocomial sepsis-attributable fractions, estimated using inversed-weight probabilities methods using generalized mixed model considering time-dependency of sepsis occurrence.

**Results:**

3588 patients from 37 hospitals were included. Mean age was 63 years and 48.8% were female at birth. 470 sepsis episodes occurred in 388 patients (311 in cases and 77 in control group), with pneumonia being the most common source of infection (44.3%). Average AF for sepsis mortality was 0.076 (95% CI 0.068–0.084) for medical admissions; 0.043 (95% CI 0.032–0.055) for elective surgical admissions; and 0.036 (95% CI 0.017–0.055) for emergency surgeries. In a time-dependent analysis, AF for sepsis rose linearly for medical admissions, reaching close to 0.12 on day 28; AF plateaued earlier for other admission types (0.04 for elective surgery and 0.07 for urgent surgery). Alternative sepsis definitions yield different estimates.

**Conclusion:**

The impact of nosocomial sepsis on outcome is more pronounced in medical admissions and tends to increase over time. The results, however, are sensitive to sepsis definitions.

**Supplementary Information:**

The online version contains supplementary material available at 10.1186/s13613-023-01123-y.

## Introduction

Sepsis is a major healthcare issue that may account for more than 11 million yearly deaths worldwide [1]. While most cases are community-acquired, nosocomial sepsis is an important source of burden for healthcare systems. Nosocomial sepsis has been repeatedly associated with increase in costs, hospital length-of-stay and mortality [2, 3], with most reports focusing on the consequences of sepsis in the population of critically ill patients [4–8], including those who were first admitted due to community-acquired sepsis [3, 6]. Attributable mortality fraction is defined as the proportion of deaths that occur related to an exposure, that is, the proportion of deaths that would not have occurred if the exposure did not take place. The attributable mortality fraction of ICU-acquired infections in critically ill patients has been suggested to be of close to 11% among patients admitted with sepsis and 21% among patients without sepsis [6], with values as high as 35% being reported for specific infections [5].

There are few reports on occurrence and impact of nosocomial infections after hospital admission in a broader population [8]. In a recent meta-analysis, only eight studies reported hospital wide data on nosocomial sepsis, but no study reported an estimate of attributable mortality fraction of nosocomial sepsis [8]. Obtaining a good estimate of attributable mortality fraction is important because it is a major driver of public health decisions [9]. Considering that overall hospital mortality is low, a case–control design may provide a good estimate of attributable mortality fraction and inform healthcare policy makers.

We conducted a case–control study aiming to estimate attributable mortality of nosocomial sepsis in Brazilian hospitals. We hypothesized that nosocomial infections would represent an important burden with high attributable mortality fraction. Due to lack of consensus on the operational definitions of nosocomial sepsis, we have also stressed data with different definitions and compared our estimates.

## Methods

### Design: observational, case–control study

#### Setting

Thirty-eight hospitals in Brazil. Hospitals in Brazil that had at least 100 beds and that had a critical care unit were eligible for participating in this study (see Additional file 1: for details). The protocol was approved at the ethics committee of the coordinating site and at all other including sites (and is available with Additional file 2). Due to the retrospective nature of the analysis, consent was waived. First patient included was admitted to the hospital in May 2018 and last patient included was admitted to the hospital in January 2020.

#### Objective

To estimate attributable mortality fraction due to nosocomial sepsis in adult patients.

#### Case and control definitions and matching

Cases were defined as hospital deaths and controls as patients who were discharged alive. After local ethics committee approval, sites were instructed to obtain a list of the most recent 50 adult patients who died during hospitalization. These patients were paired to the closest temporal patient discharged alive who had the same admission type (medical, elective surgical or urgent surgical) as the case patient. A margin of 30 days was allowed for matching. Matching was manually made; for each case, locally trained personal obtained a list for all patients discharge alive at the same day and checked for matching, if unsuccessful more medical records for a wider margin were obtained until the closest discharge alive was obtained. For example, a patient who was admitted for a medical reason and died was paired with the closest medical admission patient discharged alive from the same institution. No other matching method was performed. Elective surgical admissions were defined as admissions due to scheduled surgery. Emergency surgical admissions were defined as those whose surgery was indicated in the first 24 h after admission.

#### Sample size calculation

Sample size was calculated using Fleiss, Tytun and Ury method to estimate a difference in proportions [10]. Assuming a prevalence of sepsis in 15% in control patients, 1500 patients per group were required to have a 90% power to detect an odds ratio of at least 1.4 for the association between nosocomial sepsis and mortality. Sample size was increased to 1800 patients per group after the first protocol amendment before data were collected due to an increase in fund availability from the sponsor; this yields a similar power for an odds ratio as low as 1.35. We specified that each participating hospital would include 100 patients (50 cases and 50 controls); therefore, the increase in sample size was made by increasing the number of participating hospitals from 30 to 38, to account for possible drop-offs and incomplete pairs.

#### Data collection

Overall hospital data were collected in a structured case report form. A local data collector at each site was trained by the sponsor (HCor Research Institute) before data collection. Patient data included demographic information, comorbidities (using Charlson Comorbidity Index and Modified Frailty Index) [11, 12], reason for admission and daily data (from admission to 28 days) on suspicion of infection, antibiotic use, occurrence of organ failure (see below), and occurrence of other non-infectious clinically relevant events. The suspected infection was diagnosed at local physician’s discretion and collected from the charts. Clinically relevant events were by the steering committee on agreement, and were defined as occurrence of stroke, unstable angina or acute myocardial infarction, severe acute hypertension (hypertension that demanded medical intervention), fall, seizure, pulmonary embolism, bronchospasm, lower or upper intestinal track bleeding, and need for surgical procedure.

#### Sepsis definition

Sepsis was defined as suspected infection requiring antibiotic use plus at least one organ dysfunction in the absence of other clinically relevant events occurring in the same day. The following criteria were used for diagnosis acute organ failure: systolic blood pressure < 90 mmHg and/or mean arterial pressure < 65 mmHg and/or drop in systolic blood pressure > 40 mmHg; arterial partial pressure to inspired oxygen fraction ratio below 300 or need for supplementary oxygen to maintain peripheral oxygen saturation above 90%; abnormal mental status; increase in serum creatinine to values of at least 2 mg/dL and/or urinary output < 0.5 mL/kg/h for 2 h; total bilirubin concentration above 2 mg/dL; platelet count < 100,000 units/mm^3^; abnormal coagulation in the absence of anticoagulant use (international normalized ratio above 1.5 or activated partial thromboplastin time above 60 s). If a patient developed the before-mentioned criteria up to the second day of hospital admission, the infection was considered as not nosocomial and was therefore not considered for attributable fraction estimates; similarly, patients that were admitted with community-acquired infection and that died without having a second event within hospital admission (that is, without having nosocomial sepsis), were not considered in nosocomial sepsis-attributable fraction calculation.

### Statistical analysis

Descriptive statistics were used for univariate analyses. The primary objective was to estimate attributable fraction of mortality due to nosocomial sepsis. We assumed that in-hospital mortality would be a sufficiently rare event so that odds ratio estimates obtained from a case control design would be reliable estimators of relative risk for calculation of the nosocomial sepsis mortality attributable fraction [13–15].

The model adjustment was defined a priori, before data collection, according to the protocol available in Additional file 2; adjustment variables were elected due to clinical relevant. The following approach was used for attributable fraction calculation for each admission type: the inverted probability weights (IPW) for each patient were calculated, representing the cumulative risk of the patient acquiring sepsis during hospitalization, under a multivariable logistic regression analysis including baseline age, Charlson Comorbidity Index, and a time-dependent variable for the occurrence of clinically relevant events. We estimated the association between sepsis occurrence on hospital mortality within 28 days through a mixed logistic regression model weighted by IPW. The model included the participant center as a random intercept, and age, Charlson Comorbidity Index, infection at admission (to account for community-acquired sepsis at admission), IPW, and the accumulated dependent time variables of sepsis occurrence and clinically relevant events with their interaction with time (modeled with a third-degree polynomial). From the daily odds ratios estimated by the model, the Miettinen formula was used to calculate the attributable mortality of sepsis [16]:

$${P}_{{c}_{i}}* \frac{{\mathrm{OR}}_{i}-1 }{{\mathrm{OR}}_{i}},$$

*i* = day, *P*_c_$$ = \mathrm{proportion\,of\,patients\,that\,had\,sepsis\,among\,non-survivors}.$$

The attributable fraction assessed this way therefore represents the proportion of deaths occurring up to that day given that the patient had sepsis before that given day; that is, how the percentage of deaths that would not have occurred if the patient did not have sepsis up to that moment. We also present the marginal effect of having one sepsis episode on the whole period, with an estimate of average attributable fraction obtained from the model. All analyses were performed using the R 4.1.1 software [17]. IPW was estimated using the IPW package [18]. The delta method was used to calculate the 95% confidence intervals of the odds ratio and attributable mortality.

### Sensitivity analysis

Different definitions of sepsis were explored (Table 1); the same analysis was performed using different definitions as sensitivity analyses. An internal consensus was created within the steering committee on probability degrees of sepsis according to combinations of use of antibiotics, organ failure, results of cultures, and occurrence of clinically relevant events (Table 1; Additional file 1: Fig. S1). Two ad hoc sensitivity analyses were made, one estimating the attributable fraction considering definitive, very probable, and probable sepsis, and a second sensitivity analysis considering only definitive and very probable sepsis. This approach was used to assess whether different definitions would result in different estimates of attributable fraction. One additional post hoc analysis based on increase in Sequential Organ Failure Assessment score (SOFA) as per Sepsis 3 also performed [19, 20]; in this analysis, patients were considered to have nosocomial sepsis if their SOFA score increased at least two points over hospital admission SOFA score values and a new antibiotic was started, regardless of other information on clinically relevant events. A sensitivity analysis using the main definition but excluding patients admitted with infection was also performed. Finally, two additional sensitivity analysis were conducted for non-surgical patients adding main diagnosis category (neurological, cardiovascular, infection, renal, abdominal, or others) as predictors.

**Table 1:** Sepsis definitions used for analysis

	Antibiotic prescribed	Organ failure	Cultures	Other clinically relevant event	Sepsis source information available	Part of alternative definition 1	Part of alternative definition 2
Primary definition							
Main sepsis definition	Yes	Yes	Positive or negative	No	Yes	–	–
Alternative definition							
Definitive	Yes	Yes	Positive	No	Yes	Yes	Yes
Very probable, either:							
(1) or	Yes	Yes	Positive	Yes	Yes	Yes	Yes
(2) or	Yes	Yes	Negative	No	Yes	Yes	Yes
(3)	Yes	Yes	Negative	Yes	Yes	Yes	Yes
Probable	No	Yes	NA^a^	No	No	Yes	No
Possible	No	Yes	NA^a^	Yes	No	No	No
SOFA increase definition based on Sepsis 3 (post hoc)							
SOFA definition (“Sepsis 3”)	Yes	Yes	–		Variable	No	No

*NA* data not collected

^a^Data on cultures were only collected for patients that received antibiotics. SOFA definition was performed post hoc. Any patients that had an increase in SOFA score of at least 2 points over baseline (hospital admission) SOFA and that either were started antibiotics or received new antibiotics were considered as septic

## Results

### Hospital and patient features

A total of 1794 pairs of cases and controls from 37 hospitals were included in the analysis (one additional hospital agreed to participate but did not collect data or filled forms and was excluded from the analysis). Hospital features are shown in Additional file 1: Table S1. Six hospitals did not complete the full sample of 100 patients; in 4 cases because it was impossible to find pairs in the pre-specified timeframe. Baseline patient features are shown in Table 2. Average population age was 63 years (standard deviation of 18.6), and 48.8% were female at birth. Median Charlson Comorbidity Index was 2 (interquartile range from 0 to 4). One-fifth (22%) of all included patients had a recorded recent (in the previous month) hospital admission before the current hospital admission, and 23.7% already needed assistance for daily living activities. Median hospital length-of-stay was 8 days (interquartile range 4–14 days). Admission with acute infection occurred in 39.5% of non-survivors and 30.0% of survivors. Patient locale or status over time is shown in Additional file 1: Fig. S2. Patient features according to development or not of nosocomial sepsis are shown in Additional file 1: Table S2.

**Table 2:** Patient features, resource use and occurrence of clinically relevant events in cases and controls

	Non-survivors (*n* = 1794)	Survivors (*n* = 1794)	*p* value
Age, mean (SD)	68.5 (17.2)	57.5 (18.4)	< 0.001
Sex at birth, *n* (%)			0.095
Female	850 (47.4%)	901 (50.2%)	
Male	944 (52.6%)	893 (49.8%)	
Charlson Comorbidity Index, median [IQR]	2 [1–6]	1 [0–3]	< 0.001
Modified Frailty Index, median [IQR]	2 [1–3]	1 [0–2]	< 0.001
Previous hospitalization (last month), *n* (%)	471 (26.3%)	319 (17.8%)	< 0.001
Pneumonia on past year, *n* (%)	175 (9.8%)	79 (4.4%)	< 0.001
Episode of mental confusion on past year, *n* (%)	238 (13.3%)	116 (6.5%)	< 0.001
Previously on hospice/long-term facility/homecare, *n* (%)	93 (5.2%)	39 (2.2%)	< 0.001
Dependency for daily living activities, *n* (%)	620 (34.6%)	232 (12.9%)	< 0.001
Known comorbidities at admission, *n* (%)			
Dementia	196 (10.9%)	58 (3.2%)	< 0.001
Transitory Ischemic Attack	18 (1.0%)	18 (1.0%)	1.00
Stroke	174 (9.7%)	83 (4.6%)	< 0.001
Previous myocardial infarction	137 (7.6%)	110 (6.1%)	0.086
Angina/coronary stent	137 (7.6%)	129 (7.2%)	0.656
Heart failure	221 (12.3%)	144 (8.0%)	< 0.001
Hypertension	912 (50.8%)	767 (42.8%)	< 0.001
Diabetes, uncomplicated	473 (26.4%)	403 (22.5%)	0.007
Diabetes, complicated	136 (7.6%)	119 (6.6%)	0.299
Rheumatologic disease	66 (3.7%)	78 (4.3%)	0.349
Acquired immunodeficiency syndrome	50 (2.8%)	43 (2.4%)	0.529
Cirrhosis	65 (3.6%)	37 (2.1%)	0.006
Cancer	726 (40.5%)	453 (25.3%)	< 0.001
Hospital admission			
Admission type^a^			
Medical	1524 (84.9%)	1524 (84.9%)	–
Elective surgery	149 (8.3%)	149 (8.3%)	–
Urgent surgery/trauma	121 (6.7%)	121 (6.7%)	–
Relevant diagnosis at admission			
Infection	709 (39.5%)	538 (30.0%)	< 0.001
Respiratory diagnosis			
Asthma	11 (0.6%)	19 (1.1%)	0.199
Chronic pulmonary obstructive disease	87 (4.8%)	53 (3.0%)	0.004
Other chronic lung disease	19 (1.1%)	16 (0.9%)	0.735
Cardiac diseases			
ST-elevation myocardial infarction	31 (1.7%)	38 (2.1%)	0.466
Non-ST-elevation myocardial infarction	33 (1.8%)	28 (1.6%)	0.606
Unstable angina	18 (1.0%)	31 (1.7%)	0.083
Angina, unspecified	12 (0.7%)	9 (0.5%)	0.663
Uncompensated heart failure	137 (7.6%)	87 (4.8%)	0.001
Deep vein thrombosis	48 (2.7%)	32 (1.8%)	0.089
Pulmonary thromboembolism	27 (1.5%)	27 (1.5%)	1.00
Neurological diseases			
Ischemic stroke	83 (4.6%)	58 (3.2%)	0.039
Hemorrhagic stroke	18 (1.0%)	10 (0.6%)	0.183
Transient ischemic attack	3 (0.2%)	12 (0.7%)	0.035
Subarachnoid hemorrhage	17 (0.9%)	8 (0.4%)	0.107
Polyradiculopathy/myasthenia	3 (0.2%)	3 (0.2%)	1.00
Renal diseases			
Acute, non-related to cirrhosis	91 (5.1%)	39 (2.2%)	< 0.001
Chronic, not on dialysis	75 (4.2%)	43 (2.4%)	0.004
Chronic, needing dialysis	31 (1.7%)	40 (2.2%)	0.338
Abdominal diseases			
Uncompensated cirrhosis	36 (2.0%)	20 (1.1%)	0.042
Digestive bleeding	12 (0.7%)	7 (0.4%)	0.358
Spontaneous bacterial peritonitis	2 (0.1%)	0 (0.0%)	0.500
Hepatorenal syndrome	3 (0.2%)	0 (0.0%)	0.250
Acute pancreatitis	24 (1.3%)	27 (1.5%)	0.778
Uncompensated diabetes	70 (3.9%)	61 (3.4%)	0.477
Admission for diagnostic procedures	535 (29.8%)	515 (28.7%)	0.463
Other	648 (36.1%)	640 (35.7%)	0.808
Resource use			
Intensive care unit admission, *n* (%)	853 (47.5%)	340 (19%)	< 0.001
Hospital length-of-stay, median [IQR]	9 [5–19]	6 [4–11]	< 0.001
Antibiotic days of therapy, days per 1000 patients/day	603	437	< 0.001
Days using antibiotics, median [IQR]	5.5 [1.3–11]	1 [0–6]	< 0.001
Events during hospitalization			
Sepsis (main definition)	525 (29.3%)	232 (12.9%)	
Up to 2 days from admission	383 (21.3%)	198 (11%)	< 0.001
After 2 days from admission	311 (17.3%)	77 (4.3%)	< 0.001
Stroke	65 (3.6%)	28 (1.6%)	< 0.001
Coronary syndrome^b,c^	80 (4.5%)	42 (2.3%)	0.001
Acute severe hypertensive episode	228 (12.7%)	133 (7.4%)	< 0.001
Fall	63 (3.5%)	20 (1.1%)	< 0.001
Seizure	93 (5.2%)	34 (1.9%)	< 0.001
Pulmonary thromboembolism	40 (2.2%)	11 (0.6%)	< 0.001
Bronchospasm	118 (6.6%)	27 (1.5%)	< 0.001
Digestive bleeding	122 (6.8%)	27 (1.5%)	< 0.001
Severe pain^d^	138 (7.7%)	76 (4.2%)	< 0.001

^a^Matching variable

^b^ST-elevation myocardial infarction

^c^Non-ST-elevation myocardial infarction

^d^Pain episode that required more than 2 rescues or new diagnostic procedure

**Fig. 1:**
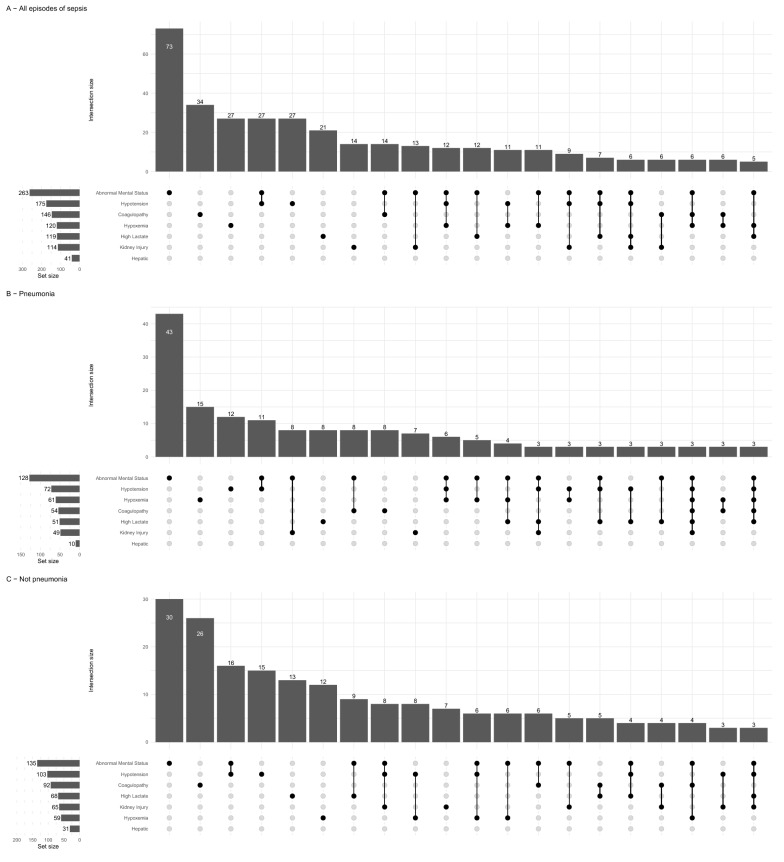
Organ failure at diagnosis in **A** all septic patients, **B** pneumonia, **C** not pneumonia. Each dot represents intersections, with the number of cases shown in bars

A total of 388 patients (311 cases and 77 control, comprising 10.8% of all included patients) had 470 nosocomial sepsis episodes (387 in cases and 83 in controls). Days of antimicrobial therapy were 603 and 437 per 1000 patients/day for cases and controls, respectively. Pneumonia was the most common source (Additional file 1: Table S3, Fig. S3). The most common organ dysfunction at sepsis diagnosis was abnormal mental status (Fig. 1). More than half of the patients had more than one of the specified organ dysfunctions during their first sepsis episode (Additional file 1: Fig. S4). Sepsis was more frequently diagnosed in ICU, followed by ward (Additional file 1: Fig. S5). There were 573 positive cultures in the population (Additional file 1: Table S4, stratified for suspected source); a list of pathogens in patients with sepsis according to the main definition is shown in Additional file 1: Table S5 (74 patients with any positive culture).

### Attributable mortality fraction

**Fig. 2:**
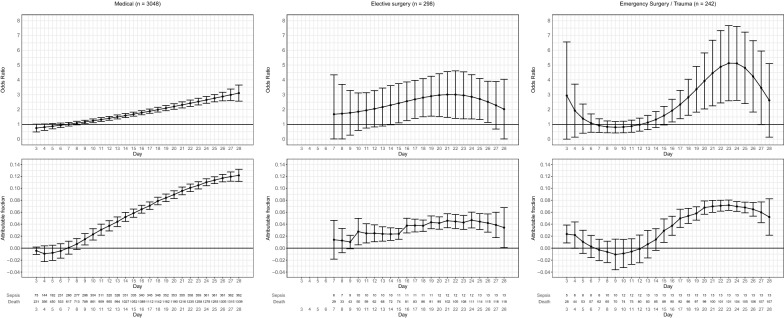
Distribution of odds ratio (upper row) and AF (lower row) according to admission type (columns). The odds ratio should be interpreted as the odds ratio of dying up to a specific day (*x*-axis) given that the patient has acquired sepsis

Daily odds ratio for mortality obtained from the model and its respective attributable fraction are shown in Fig. 2. The reported odds ratio is the effect size of dying up to a specific day (x-axis) given the patient had infection in the preceding days (up to hospital admission). Attributable fraction is interpreted as percentage of deaths occurring up to that day given that the patient previously had sepsis. For medical admissions, attributable fraction rose after the sixth day of admission, and reached close to 0.12 at 28 days. The effect of nosocomial sepsis was less pronounced on elective surgery and urgent surgery patients, with peak values around 0.04–0.07, and a less linear increase over time.

Overall marginal odds ratio for mortality and attributable fraction for patients with any sepsis episode were 1.73 (95% CI 1.60; 1.87), attributable fraction = 0.076 (95% CI 0.068; 0.084); 2.75 (95% CI 1.47; 4.03), attributable fraction = 0.043 (95% CI 0.032; 0.055); and 1.75 (95% CI 1.06; 2.43), attributable fraction = 0.036 (95% CI 0.017; 0.055]), for medical, elective surgery and emergency surgery patients, respectively.

### Alternative analysis 1: definitive, very probable and probable sepsis

Using this broader definition, a total of 1129 patients had nosocomial sepsis during hospital stay. A total of 1387 septic episodes were recorded (1058 on non-survivors and 329 in survivors). 74 patients had definitive sepsis, 850 very probable sepsis and 205 probable sepsis (see Additional file 1: Fig. S6 for details on infection source of organ dysfunction). The overall marginal odds ratio for mortality and attributable fraction for patients with one episode of definite, very probable, or probable sepsis were 1.32 (95% CI 1.25; 1.39), attributable fraction = 0.101 (95% CI 0.083; 0.118); 2.85 (95% CI 2.32; 3.39), attributable fraction = 0.374 (95% CI 0.337; 0.412); and 2.21 (95% CI 1.77; 2.64), attributable fraction = 0.230 (95% CI 0.192; 0.268) for medical, elective surgery and emergency surgery groups. Results over time are shown Additional file 1: Fig. S7; this definition resulted in an attributable fraction of up to 0.25 for medical admissions, and higher values for elective and urgent surgery than the main definition used (peaking over 0.45 at 28 days for elective surgery), with a more linear ascend over time.

### Alternative analysis 2: definitive and very probable sepsis

A total of 924 patients had nosocomial sepsis as defined using the definitive and very probable sepsis criteria, totalizing 1121 septic episodes (945 on non-survivors and 176 in survivors (Additional file 1: Fig. S8 shows infection sources and organ dysfunctions). Marginal odds ratio for mortality and attributable fraction under this definition were 1.60 (95% CI 1.51; 1.70), attributable fraction = 0.141 (95% CI 0.128; 0.155); 3.61 (95% CI 2.90; 4.32, attributable fraction = 0.392 (95% CI 0.363; 0.422); and 2.57 (95% CI 2.04; 3.10), attributable fraction = 0.251 (95% CI 0.218; 0.284), for medical, elective surgical and emergency surgery groups. Results for odds ratio and attributable fraction over time are shown in Additional file 1: Fig. S9.

### Post hoc analysis: SOFA definition (based on Sepsis 3)

729 patients had nosocomial sepsis as by an increase in SOFA score of at least two points over baseline (869 septic episodes: 782 on non-survivors and 87 in survivors). Marginal odds ratio for mortality and attributable fraction under this definition were 2.25 (95% CI 2.10; 2.39), attributable fraction = 0.176 (95% CI 0.167; 0.185); 3.52 (95% CI 2.82; 4.22, attributable fraction = 0.358 (95% CI 0.330; 0.386); and 2.93 (95% CI 2.30; 3.56), attributable fraction = 0.246 (95% CI 0.219; 0.274), for medical, elective surgical and emergency surgery groups. Results for odds ratio and attributable fraction over time are shown in Additional file 1: Fig. S10.

### Post hoc analysis: exclusion of patients admitted with infection under the main definition

A total of 3007 patients were considered in this analysis—1411 non-survivors and 1596 survivors—with a total of 177 and 37 episodes of sepsis, respectively. Odds ratio for mortality and attributable fraction were 2.21 (95% CI 1.95; 2.47), attributable fraction = 0.055 (95% CI 0.05; 0.061); 2.84 (95% CI 1.35; 4.31, attributable fraction = 0.034 (95% CI 0.024; 0.043); and 1.71 (95% CI 0.94; 2.48), attributable fraction = 0.035 (95% CI 0.013; 0.057), for medical, elective surgical and emergency surgery groups (Additional file 1: Fig. S11).

### Post hoc analysis: additional adjustment for main reason for admission using main definition and the SOFA definition for non-surgical patients

Adding further adjustment according to main admission category for non-surgical patients analyses yield an average odds ratio for mortality of 1.64 (95% CI 1.51; 1.76) with an average attributable fraction of 0.070 (95% CI of 0.061; 0.0782); time-dependent effects are shown in Additional file 1: Fig. S12. The only admission type that was consistently associated with an attributable fraction above 0.10 was admission due to infection (Additional file 1: Fig. S13), reinforcing the importance of adjustment for infection in the primary analysis. For the increase SOFA definition (Sepsis 3), adding admission category resulted in average odds ratio was 2.39 (95% CI 2.24; 2.54) and average attributable fraction was 0.184 (95% CI 0.176; 0.192).

### Comparison of the definitions

**Fig. 3:**
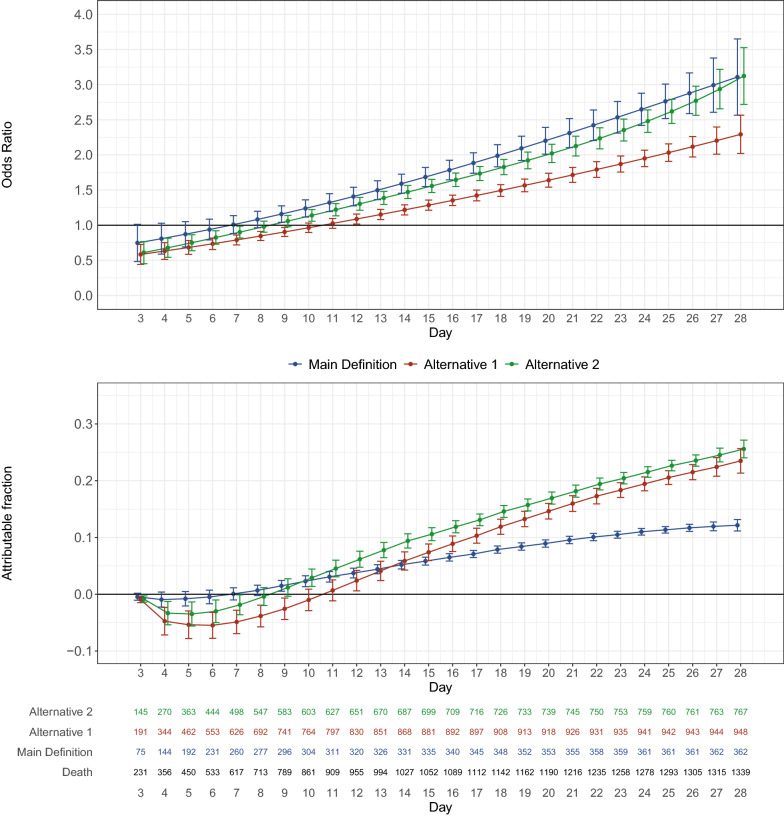
Comparison of the three definitions for medical admissions. Upper panel: the daily odds ratio for mortality in medical patients according to the definition used. Bottom panel: the respective AF for each definition. Note that a higher OR does not equal higher AF due to changes in prevalence

Results for the comparison of the odds ratio and attributable fraction for medical admission are shown in Fig. 3. Odds ratio for mortality increased over time for all definitions; however, due to differences in prevalence of events, the attributable fraction was lower for the main definition when compared to both alternative definitions. Comparison for the definitions for elective surgery and emergency surgery patients is shown in Additional file 1: Fig. S14 and S15; the main definition provided the lower AF for surgical patients. The post hoc analysis based on SOFA score increase as per Sepsis 3 suggestion provided resulted in estimates for odds ratio and attributable fraction that largely followed the results of the second alternative definition. Exclusion of patients with known sepsis at admission reduced the attributable fraction of nosocomial sepsis due to decrease in number of events (Additional file 1: Fig. S11). Adding further adjustments for admission type also did not change estimates for non-surgical patients (Additional file 1: Table S6).

## Discussion

In this case–control study including 1794 pairs of patients from 37 Brazilian hospitals, we found that nosocomial sepsis, defined by an acute nosocomial infection with organ failure in the absence or other clinically relevant events, was an important contributor to hospital mortality with a significant attributable mortality fraction. Under this definition, attributable fraction was higher for medical admissions than for elective surgical and emergency surgical patients. Attributable fraction over time linearly increased up to 28 days for medical patients, but not for elective and emergency surgery admissions where a plateau was observed. Pneumonia was the most common infection source, and abnormal mental status was the commonest organ dysfunction observed. Antibiotic use was high in both groups.

Different sepsis definitions will inevitably be associated with different prevalence and effect sizes for mortality, with consequential direct impact on attributable mortality fraction. We also explored additional definitions of sepsis according to key features including positive cultures, occurrence of clinically relevant events, and antibiotic use. The fact that clinically relevant events, obtained directly from chart review, was considered before attributing organ failure to infection may enhance the capability of measuring the effect associated directly with infection. Indeed, organ failure is associated with a myriad of clinical conditions [21]; this creates a situation where antibiotics are prescribed due to new organ failure even if coexisting events that may be responsible for organ failure occur simultaneously. Our main definition was stricter than other sepsis definitions by limiting sepsis diagnosis to the absence of coexisting events that could cause organ failure at the same day [20]. The two alternative definitions were more comprehensive, as seen by the higher number of cases reported: the first being broader than the second. The first alternative attributed new organ dysfunctions in the absence of clinically relevant events as septic events even in the absence of antibiotic use (“any new organ dysfunction in a hospitalized patient is sepsis until proven otherwise”), while the second did not include such patients. The resulting attributable fractions reflected the changes in both effect size (odds ratio) and prevalence; despite being associated with higher odds ratios for mortality, the main definition had the lowest attributable fraction. Differences in attributable fraction among definitions were specially pressing in surgical patients. A definition based on increase in SOFA provided results similar to the second alternative definition. When baseline sepsis patients were excluded, the attributable fraction was markedly reduced (which is expected since infection is an important risk factor for a secondary insult), but the odds ratio for mortality remained high.

These findings have several important implications. First, even under the strictest definition sepsis-attributable fraction was still very important, reaching around 0.12 for prolonged medical admissions; the longer the medical patient remained in the hospital, the highest the odds ratio for mortality associated with a septic episode. This value is aligned with other estimated of attributable fraction in more severe patients and may represent a reasonable starting point for quality improvement initiatives [5]. These results, under the main definition, should probably be seen as a minimum value for sepsis-attributable fraction in hospitalized patients. Albeit important, an average attributable fraction ranging from 0.036 (emergency surgical patients) to 0.076 (medical patients) is sufficiently small so that an intervention may reduce nosocomial infections without easily noticeable effects in mortality, unless sample size is very large, under this definition. Since infection burden is not exclusively related to mortality, involving costs, long-term outcomes, quality of life, among others, understanding sepsis-attributable fraction may avoid over-simplistic conclusions, such as interpreting that an intervention that reduces infection occurrence but not mortality prevented only non-fatal infections or even that some infections may not be associated with higher mortality at all.

The variability observed by tweaking sepsis definitions should also be seen an alert that it is hard to isolate the impact of a single event within the intricate path of a hospital stay, specifically for surgical patients where different broader definitions provided results strikingly different from medical patients. It is conceivable that “true” sepsis-attributable fraction may be somewhere in between the main definition and the second alternative definition. Far from suggesting that nosocomial sepsis is not an issue, our results highlight that even when considering known factors for poor hospital outcome such as age and comorbidities, sepsis could be directly responsible from something between 7.6% and 14.1% of all hospital death for medically admitted patients. For surgical patients, the margins are even wider depending on the definition used, peaking over 40%. Attributable fraction is a relative measurement, and not a direct estimate of burden. Low-middle income countries are suggested to be more affected by nosocomial sepsis, which may result in a higher numeric burden of deaths in this population.

We hope that this manuscript fosters the discussion on whether sepsis would benefit from a more nuanced diagnosis approach where probability categories are used to tailor diagnosis and treatment (as is the case of aspergillosis, where possible/probable categories have been in use but may also be applicable to other medical conditions) [22, 23]. Despite over 30 years of controversy, all sepsis definitions are *binary*, that is, they do not consider the uncertainty that permeates clinical decision-making, focusing more on severity of illness than in the very probability that the findings are due to active infection.

Our work has several limitations. As with any case control trial, selecting appropriate controls is challenging. We used the closest temporal admission discharged alive with the same admission type as this seemed a good compromise between feasibility and adequacy. As can be seen in Table 1, the number of possible clinical conditions is very large, and if matching criteria were too strict, we would have ended with issues in obtaining proper controls; we tried to overcome this by adjusting for several relevant confounders, including age, comorbidities, etc. Most hospitals in this study still used non-electronic (paper) healthcare records, thereby making triaging of possible controls challenging. Restricting our study to only hospitals with electronic healthcare records would induce another source of bias, since these hospitals would inherently have more resources. We estimated attributable fraction from a case–control study, which is also limited by the assumption that odds ratio and relative risk will be similar when outcomes are infrequent. This is, in fact, the de facto approach made by several statistical packages that estimate the attributable fraction [24]. Our model adjusted for time-dependency of covariates but did not consider further daily information besides diagnosis of infection and clinically relevant events. The main adjustment model was defined a priori, according to a stablished protocol. All variable selection approaches may be subject to criticism, but we refrained from using variables that were associated with either sepsis or mortality as suggested [25]. Adding admission categories to the main analysis did not change the estimated of AF significantly. Other approaches could be employed to estimate attributable fraction [26]. The list of clinically relevant events is not exhaustive and was defined by the steering committee during protocol discussions before data were collected but is somewhat arbitrary. Finally, our results reflect the Brazilian panorama; it is uncertain whether they are transposable to other settings.

## Conclusion

Nosocomial sepsis is an important contributor to hospital mortality. The impact of nosocomial sepsis on outcome is more pronounced in medical admissions and tends to increase over time. Different sepsis definitions led to important changes on attributable fraction.

## Supplementary Information

**Additional file 1**: **Table S1**. Hospital Features. **Table S2**. Comparison between patients that develop and did not develop nosocomial sepsis according to the main definition. **Table S3**. Sepsis episodes. **Figure S1**. Venn diagram for the sepsis definitions used in the manuscript. **Figure S2**. Daily patient location/status over time for controls (top) and cases (bottom). **Figure S3**. Number of nosocomial sepsis episodes according to infection source. **Figure S4**. Number of organ dysfunctions at presentation for (A) first episode of sepsis, and (B) all sepsis episodes. **Figure S5**. Patient locale at sepsis diagnosis. Top: Stratified according to outcome; Bottom: All patients. **Table S4**. Pathogens isolated from cultures according to suspected site; more than one pathogen was possible for each patient. CRBI: Catheter related bloodstream infections. Note that pathogens could be isolated in any culture collected from the patient within the 48h timeframe. The local diagnosis was considered as reference; therefore the isolated pathogen could not be considered the culprit for the infection. **Table S5**. Positive cultures for patients that had one septic episode according to main definition. CRBI: Catheter related bloodstream infections. Note that pathogens could be isolated in any culture collected from the patient within the 48h timeframe. Same as in Table S3, the final diagnosis was made by the site. **Figure S6**. (A) Infection source considering first alternative definition. The number of patients that did not receive antibiotic reflects patients that developed new organ failure in the absence of any other clinically relevant event and were considered as possibly septic under this definition. (B) Organ dysfunction at presentation for the alternative definition 1 analysis. **Figure S7**. Distribution of odds ratio (upper row) and AF (lower row) according to admission type (columns) for the first alternative definition considering the effects of definitive, very probably, and probable sepsis. **Figure S8**. Sepsis sources (A) and organ dysfunction at presentation for the second alternative definition analysis. **Figure S9**. Distribution of odds ratio (upper row) and PAF (lower row) according to admission type (columns) for the second alternative definition considering the effects of definitive and very probably sepsis. **Figure S10**. Distribution of odds ratio (upper row) and PAF (lower row) according to admission type (columns) for the post hoc definition based on SOFA score. **Figure S11**. Distribution of odds ratio (upper row) and PAF (lower row) according to admission type (columns) after excluding patients with infection at baseline. **Figure S12.** Comparison of the three definitions for elective surgery admissions. Upper panel: The daily odds ratio for mortality in elective surgery patients according to the definition used. Bottom panel: The respective AF for each definition. Note that a higher OR does not equal higher AF since prevalence of events also changes. **Figure S13**. Comparison of the three definitions for emergency surgery admissions. Upper panel: The daily odds ratio for mortality in medical patients according to the definition used. Bottom panel: The respective AF for each definition. Note that a higher OR does not equal higher AF since prevalence of events also changes.

**Additional file 2**: IMPACTO-MAPA study.

## Data Availability

De-identified data, data dictionary, and analyses scripts used are available upon reasonable request and after approval of the steering committee of the proposal and after agreement of Brazilian Ministry of Health for data sharing.
