# Supplementary material for: Attributable mortality due to nosocomial sepsis in Brazilian hospitals: a case–control study
Source: Ann Intensive Care. 2023 Apr 26;13:32. doi: 10.1186/s13613-023-01123-y (PMC10133434; doi:10.1186/s13613-023-01123-y)
Supplement: Supplementary file 1 — Additional file 1: Table S1. Hospital Features. Table S2. Comparison between patients that develop and did not develop nosocomial sepsis according to the main definition. Table S3. Sepsis episodes. Figure S1. Venn diagram for the sepsis definitions used in the manuscript. Figure S2. Daily patient location/status over time for controls (top) and cases (bottom). Figure S3. Number of nosocomial sepsis episodes according to infection source. Figure S4. Number of organ dysfunctions at presentation for (A) first episode of sepsis, and (B) all sepsis episodes. Figure S5. Patient locale at sepsis diagnosis. Top: Stratified according to outcome; Bottom: All patients. Table S4. Pathogens isolated from cultures according to suspected site; more than one pathogen was possible for each patient. CRBI: Catheter related bloodstream infections. Note that pathogens could be isolated in any culture collected from the patient within the 48h timeframe. The local diagnosis was considered as reference; therefore the isolated pathogen could not be considered the culprit for the infection. Table S5. Positive cultures for patients that had one septic episode according to main definition. CRBI: Catheter related bloodstream infections. Note that pathogens could be isolated in any culture collected from the patient within the 48h timeframe. Same as in Table S3, the final diagnosis was made by the site. Figure S6. (A) Infection source considering first alternative definition. The number of patients that did not receive antibiotic reflects patients that developed new organ failure in the absence of any other clinically relevant event and were considered as possibly septic under this definition. (B) Organ dysfunction at presentation for the alternative definition 1 analysis. Figure S7. Distribution of odds ratio (upper row) and AF (lower row) according to admission type (columns) for the first alternative definition considering the effects of definitive, very probably, and probable sepsis. Figure [file 13613_2023_1123_MOESM1_ESM.docx]

**Attributable mortality due to nosocomial sepsis in Brazilian hospitals: A case-control study**

**Supplementary Material**

**Table of Contents**

[**eTable 1** – Hospital Features 4](#_Toc126864339)

[**eTable 2 –** Comparison between patients that develop and did not develop nosocomial sepsis according to the main definition 5](#_Toc126864340)

[**eTable 3** – Sepsis episodes. 7](#_Toc126864341)

[**eFigure 1** – Venn diagram for the sepsis definitions used in the manuscript 8](#_Toc126864342)

[**eFigure 2** – Daily patient location/status over time for controls (top) and cases (bottom) 9](#_Toc126864343)

[**eFigure 3 -** Number of nosocomial sepsis episodes according to infection source 10](#_Toc126864344)

[**eFigure 4** – Number of organ dysfunctions at presentation for (A) first episode of sepsis, and (B) all sepsis episodes. 11](#_Toc126864345)

[**eFigure 5** – Patient locale at sepsis diagnosis. Top: Stratified according to outcome; Bottom: All patients. 12](#_Toc126864346)

[**eTable 4 –** Pathogens isolated from cultures according to suspected site; more than one pathogen was possible for each patient. CRBI: Catheter related bloodstream infections. Note that pathogens could be isolated in any culture collected from the patient within the 48h timeframe. The local diagnosis was considered as reference; therefore the isolated pathogen could not be considered the culprit for the infection. 13](#_Toc126864347)

[**eTable 5 –** Positive cultures for patients that had one septic episode according to main definition. CRBI: Catheter related bloodstream infections. Note that pathogens could be isolated in any culture collected from the patient within the 48h timeframe. Same as in eTable 3, the final diagnosis was made by the site. 14](#_Toc126864348)

[**eFigure 6** – (A) Infection source considering first alternative definition. The number of patients that did not receive antibiotic reflects patients that developed new organ failure in the absence of any other clinically relevant event and were considered as possibly septic under this definition. (B) Organ dysfunction at presentation for the alternative definition 1 analysis. 15](#_Toc126864349)

[**eFigure 7** – Distribution of odds ratio (upper row) and AF (lower row) according to admission type (columns) for the first alternative definition considering the effects of definitive, very probably, and probable sepsis. 16](#_Toc126864350)

[**eFigure 8** – Sepsis sources (A) and organ dysfunction at presentation for the second alternative definition analysis. 17](#_Toc126864351)

[**eFigure 9** – Distribution of odds ratio (upper row) and PAF (lower row) according to admission type (columns) for the second alternative definition considering the effects of definitive and very probably sepsis. 18](#_Toc126864352)

[**eFigure 10 -** Distribution of odds ratio (upper row) and PAF (lower row) according to admission type (columns) for the post-hoc definition based on SOFA score 19](#_Toc126864353)

[**eFigure 11 -** Distribution of odds ratio (upper row) and PAF (lower row) according to admission type (columns) after excluding patients with infection at baseline. 20](#_Toc126864354)

[**eFigure 12 –** Comparison of the three definitions for elective surgery admissions. Upper panel: The daily odds ratio for mortality in elective surgery patients according to the definition used. Bottom panel: The respective AF for each definition. Note that a higher OR does not equal higher AF since prevalence of events also changes. 21](#_Toc126864355)

[**eFigure 13** – Comparison of the three definitions for emergency surgery admissions. Upper panel: The daily odds ratio for mortality in medical patients according to the definition used. Bottom panel: The respective AF for each definition. Note that a higher OR does not equal higher AF since prevalence of events also changes. 22](#_Toc126864356)

**Supplemental Methods**

Hospital Selection

Hospital selection was not random, that is, we did not use a random sampling of hospitals from a list of all hospitals in Brazil, such as performed in the SPREAD study. We invited hospitals that had any previous relationship with Hcor research institute (i.e., previous participation in any clinical study coordinated by our institution, mostly the CHECKLIST ICU Trial and the BaSICS trial [1,2]) and those that manifested interest in participating in the study during regular BRICNet meetings. Due to requirements from Brazilian Ministry of Health, hospital from all 5 macro regions in Brazil were invited. Sites received a feasibility questionnaire, and the sites were selected based on chronological order (“first come, first served”). If one site was unable to participate or declined participation after an initial acceptance, we moved to the next site in the waiting list.

References:

1 Cavalcanti AB, Bozza FA, Machado FR, et al. Effect of a Quality Improvement Intervention With Daily Round Checklists, Goal Setting, and Clinician Prompting on Mortality of Critically Ill Patients: A Randomized Clinical Trial. *JAMA* 2016; **315:** 1480–90.

2 Zampieri FG, Machado FR, Biondi RS, et al. Effect of Intravenous Fluid Treatment With a Balanced Solution vs 0.9% Saline Solution on Mortality in Critically Ill Patients: The BaSICS Randomized Clinical Trial. *JAMA* 2021; **326:** 1–12.

# **eTable 1** – Hospital Features

| Features | Total (n=37) |
| --- | --- |
| General Features | |
| Majority of beds used for private insurance holders | 15/37 (40.5%) |
| Major source of funding |  |
| Public | 11/37 (29.7%) |
| Private, for profit | 19/37 (51.4%) |
| Private, philanthropic | 7/37 (18.9%) |
| University affiliated | 12/37 (32.4%) |
| Accreditation |  |
| National, level I | 1/37 (2.7%) |
| National, level II | 4/37 (10.8%) |
| National, level III | 4/37 (10.8%) |
| JCI | 6/37 (16.2%) |
| Canadian | 2/37 (5.4%) |
| Other | 3/37 (8.1%) |
| None | 17/37 (45.9%) |
| Number of beds |  |
| Hospital; median [IQR] | 223 [158 - 338] (n=37) |
| ICU; median [IQR] | 28 [17 - 36] (n=37) |
| Emergency Department |  |
| No | 5/37 (13.5%) |
| Referenced only | 10/37 (27%) |
| Open | 22/37 (59.5%) |
| Operational and Staffing Features | |
| Number of beds per nurse on wards; median [IQR] | 17 [12 - 22] (n=37) |
| Rapid Response Team Available | 23/37 (62.2%) |
| Institutional Protocols |  |
| Sepsis | 23/37 (62.2%) |
| Sedation on ICU | 18/37 (48.6%) |
| Myocardial Infarction | 19/37 (51.4%) |
| Mechanical Ventilation | 20/37 (54.1%) |
| Stroke | 17/37 (45.9%) |
| Other protocols | 17/37 (45.9%) |
| ICU |  |
| Waiting list for ICU at least once a week* | 17/37 (45.9%) |
| ICU Waiting list more than 2 days a week* | 16/17 (94.1%) |
| Beds/physician; median [IQR] | 10 [8 - 10] (n=37) |
| Beds/nurse; median [IQR] | 6 [5 - 10] (n=37) |
| Beds/Nurse Assistant; median [IQR] | 2 [2 - 2] (n=37) |

* Waiting list refers to patients on queue for ICU admission due to unavailability of beds.

# **eTable 2 –** Comparison between patients that develop and did not develop nosocomial sepsis according to the main definition

|  | **No** | **Yes** | **Total** | **p** |
| --- | --- | --- | --- | --- |
|  | **(n=3200)** | **(n=388)** | (n=3588) |  |
| **Age, mean (SD)** | 62.4 (18.4) | 68 (19.4) | 63 (18.6) | <0,001 |
| **Sex at birth, n (%)** | |  |  |  |
| Female | 1562 (48.8%) | 189 (48.7%) | 1751(48.8%) | 1 |
| Male | 1638 (51.2%) | 199 (51.3%) | 1837 (51.2%) |  |
| **Charlson Comorbidity Index, median [IQR]** | 2 [0 - 4] | 1 [0 - 2] | 2 [0 - 4] (n=3588) | <0,001 |
| **Modified Frailty Index, median [IQR]** | 1 [0 - 2] | 2 [1 - 3] | 1 [0 - 2] (n=3588) | <0,001 |
| **SOFA at baseline, median [IQR]** | 1 [0 - 3] | 2 [0 - 5] | 1 [0 - 3] (n=3588) | <0,001 |
| **Previous hospitalization (last month), n (%)** | 719 (22.5%) | 71 (18.3%) | 790 (22%) | 0.069 |
| **Pneumonia on past year, n (%)** | 217 (6.8%) | 37 (9.5%) | 254 (7.1%) | 0.058 |
| **Episode of mental confusion on past year, n (%)** | 307 (9.6%) | 47 (12.1%) | 354 (9.9%) | 0.125 |
| **Previously on hospice/long term facility/homecare, n (%)** | 106 (3.3%) | 26 (6.7%) | 132 (3.7%) | 0.002 |
| **Dependency for daily living activities, n (%)** | 724 (22.6%) | 128 (33%) | 852 (23.7%) | <0,001 |
| **Known comorbidities at admission, n (%)** | | |  |  |
| Dementia | 207 (6.5%) | 47 (12.1%) | 254 (7.1%) | <0,001 |
| Transitory Ischemic Attack | 34 (1.1%) | 2 (0.5%) | 36 (1%) | 0.424 |
| Stroke | 212 (6.6%) | 45 (11.6%) | 257 (7.2%) | 0.001 |
| Previous Myocardial Infarction | 218 (6.8%) | 29 (7.5%) | 247 (6.9%) | 0.596 |
| Angina/Coronary Stent | 237 (7.4%) | 29 (7.5%) | 266 (7.4%) | 0.919 |
| Heart Failure | 293 (9.2%) | 72 (18.6%) | 365 (10.2%) | <0,001 |
| Hypertension | 1489 (46.5%) | 190 (49%) | 1679 (46.8%) | 0.389 |
| Diabetes, uncomplicated | 771 (24.1%) | 105 (27.1%) | 876 (24.4%) | 0.211 |
| Diabetes, complicated | 226 (7.1%) | 29 (7.5%) | 255 (7.1%) | 0.754 |
| Rheumatologic Disease | 125 (3.9%) | 19 (4.9%) | 144 (4%) | 0.338 |
| Acquired Immunodeficiency Syndrome | 72 (2.2%) | 21 (5.4%) | 93 (2.6%) | 0.001 |
| Cirrhosis | 80 (2.5%) | 22 (5.7%) | 102 (2.8%) | 0.002 |
| **Hospital Admission** | | |  |  |
| **Admission Type*** | | |  | |
| Medical | 2686 (83.9%) | 362 (93.3%) | 3048 (84.9%) | <0,001 |
| Elective Surgery | 285 (8.9%) | 13 (3.4%) | 298 (8.3%) |  |
| Urgent Surgery/Trauma | 229 (7.2%) | 13 (3.4%) | 242 (6.7%) |  |
| **Relevant diagnosis at admission** | | |  | |
| Infection | 1032 (32.2%) | 215 (55.4%) | 1247 (34.8%) | <0,001 |
| Respiratory Diagnosis | | |  | |
| Asthma | 25 (0.8%) | 5 (1.3%) | 30 (0.8%) | 0.367 |
| COPD | 113 (3.5%) | 27 (7%) | 140 (3.9%) | 0.002 |
| Other chronic lung disease | 24 (0.8%) | 11 (2.8%) | 35 (1%) | 0.001 |
| Cardiac Diseases | |  |  |  |
| STEMI | 65 (2%) | 4 (1%) | 69 (1.9%) | 0.238 |
| UNSTEMI | 52 (1.6%) | 9 (2.3%) | 61 (1.7%) | 0.299 |
| Unstable angina | 47 (1.5%) | 2 (0.5%) | 49 (1.4%) | 0.163 |
| Angina, unspecified | 19 (0.6%) | 2 (0.5%) | 21 (0.6%) | 1 |
| Uncompensated Heart Failure | 187 (5.8%) | 37 (9.5%) | 224 (6.2%) | 0.007 |
| Deep Vein Thrombosis | 74 (2.3%) | 6 (1.5%) | 80 (2.2%) | 0.465 |
| Pulmonary Thromboembolism | 50 (1.6%) | 4 (1%) | 54 (1.5%) | 0.514 |
| Neurological Diseases | |  |  |  |
| Ischemic Stroke | 127 (4%) | 14 (3.6%) | 141 (3.9%) | 0.89 |
| Hemorrhagic Stroke | 27 (0.8%) | 1 (0.3%) | 28 (0.8%) | 0.356 |
| Transient ischemic attack | 15 (0.5%) | 0 (0%) | 15 (0.4%) | 0.395 |
| Subarachnoid Hemorrhage | 21 (0.7%) | 4 (1%) | 25 (0.7%) | 0.339 |
| Polyradiculopathy /Myasthenia | 5 (0.2%) | 1 (0.3%) | 6 (0.2%) | 0.497 |
| Abdominal Diseases | |  |  |  |
| Uncompensated Cirrhosis | 40 (1.2%) | 16 (4.1%) | 56 (1.6%) | <0,001 |
| Digestive bleeding | 15 (0.5%) | 4 (1%) | 19 (0.5%) | 0.142 |
| Spontaneous bacterial peritonitis | 1 (0%) | 1 (0.3%) | 2 (0.1%) | 0.205 |
| Hepatorenal syndrome | 3 (0.1%) | 0 (0%) | 3 (0.1%) | 1 |
| Acute Pancreatitis | 49 (1.5%) | 2 (0.5%) | 51 (1.4%) | 0.167 |
| Uncompensated diabetes | 120 (3.8%) | 11 (2.8%) | 131 (3.7%) | 0.472 |
| Admission for diagnostic procedures | 919 (28.7%) | 131 (33.8%) | 1050 (29.3%) | 0.044 |
| Other | 1180 (36.9%) | 108 (27.8%) | 1288 (35.9%) | <0,001 |

# **eTable 3** – Sepsis episodes.

|  | Non-survivors  N=1794 | Survivors  N=1794 | All Patients |
| --- | --- | --- | --- |
| Patients with at least one septic episode | 311(17.3%) | 77(4.3%) | 388 (10.8%) |
| Total number of septic episodes | 387 | 83 | 470 |
| Frequency of septic episodes by source* |  |  |  |
| Pneumonia | 185 (47.8%) | 23 (27.7%) | 208 (44.3%) |
| Unknown/Unclear | 77 (19.9%) | 21 (25.3%) | 98 (20.9%) |
| Urinary | 38 (9.8%) | 8 (9.6%) | 46 (9.8%) |
| Other | 46 (11.9%) | 11 (13.3%) | 57 (12.3%) |
| Abdominal | 21 (5.4%) | 10 (12.1%) | 31 (6.6%) |
| Skin/Soft Tissue | 20 (5.2%) | 10 (12.1%) | 30 (6.4%) |

* Percentages are for total number of episodes

# **eFigure 1** – Venn diagram for the sepsis definitions used in the manuscript


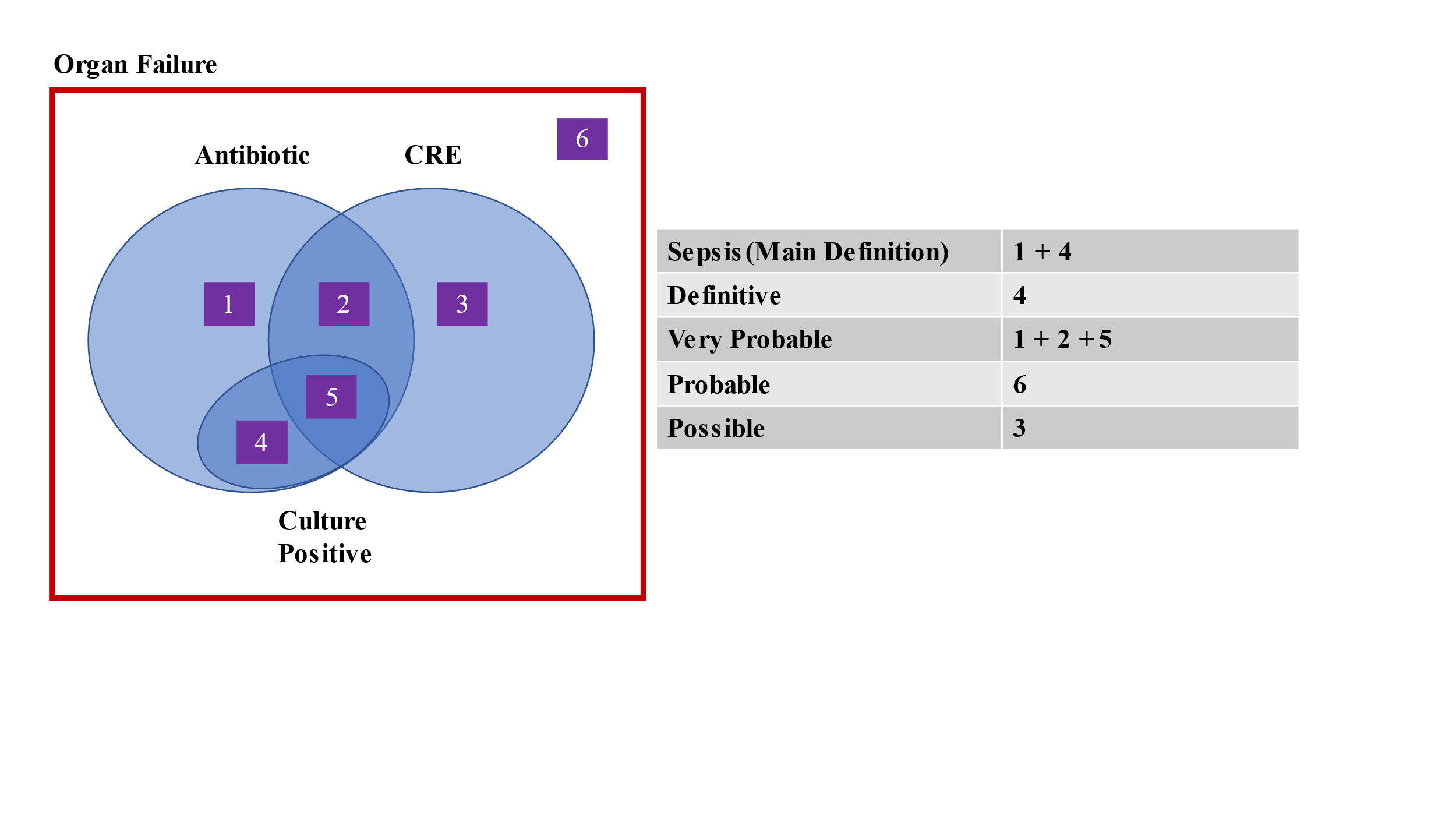


# **eFigure 2** – Daily patient location/status over time for controls (top) and cases (bottom)


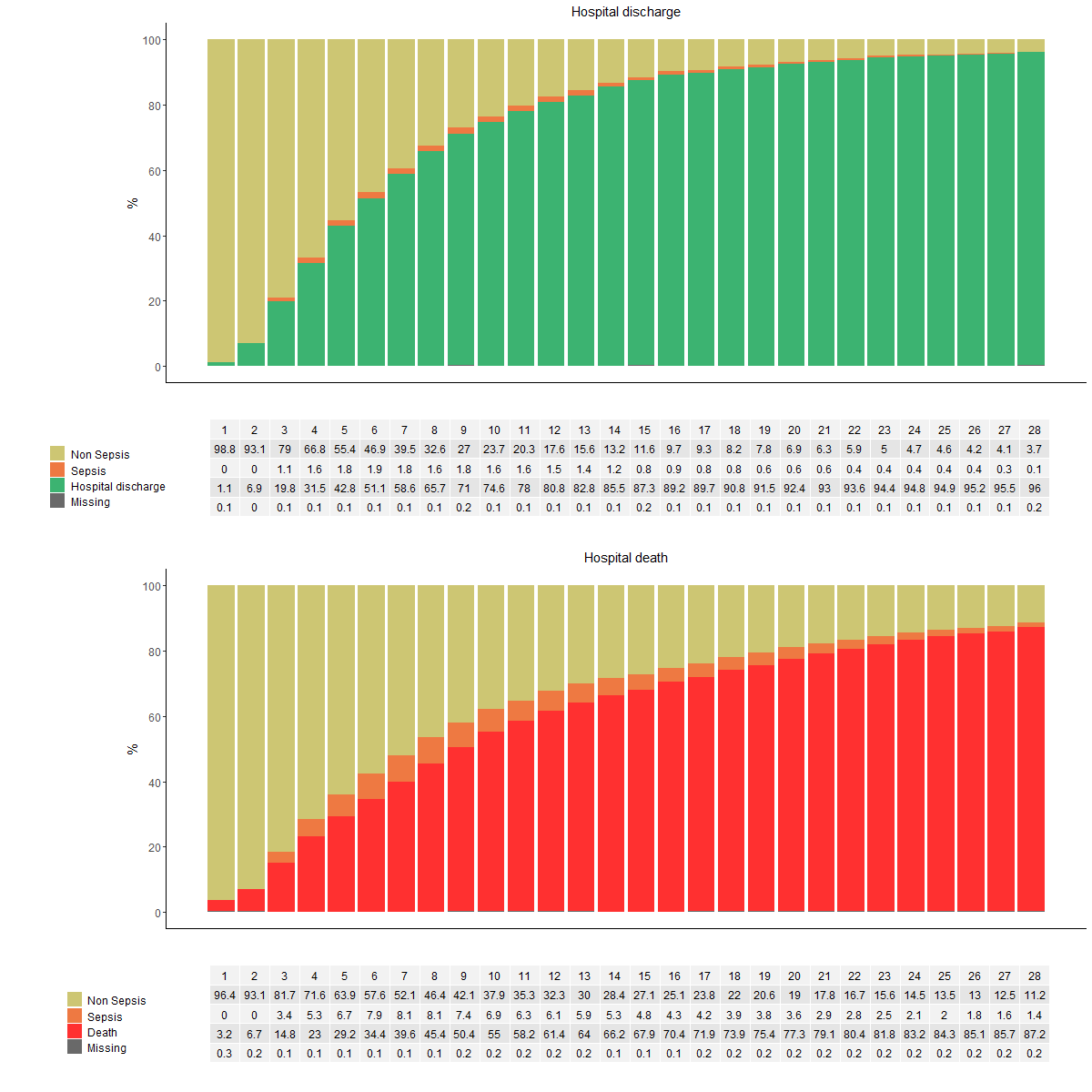


# **eFigure 3 -** Number of nosocomial sepsis episodes according to infection source

**
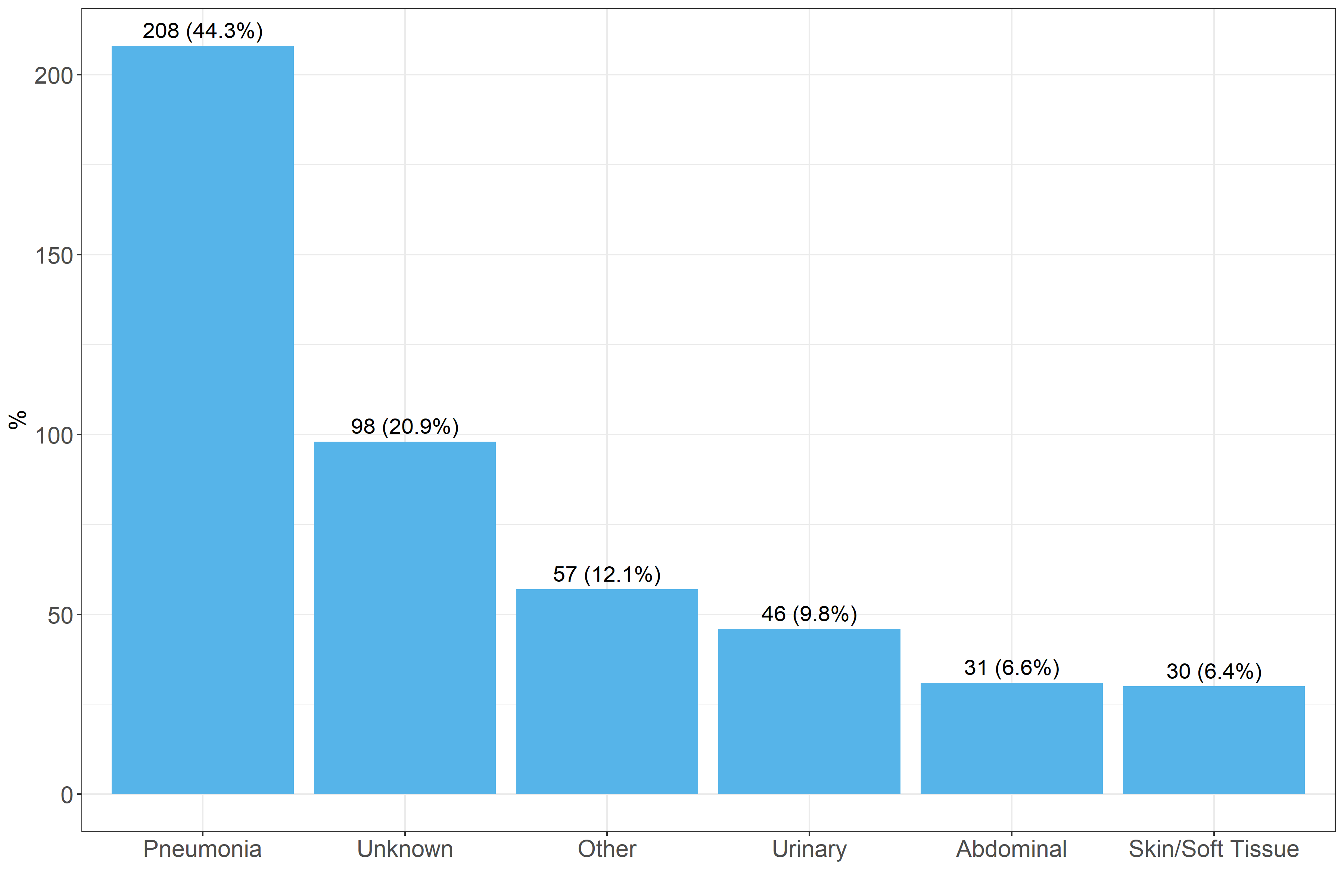
**

# **eFigure 4** – Number of organ dysfunctions at presentation for (A) first episode of sepsis, and (B) all sepsis episodes.


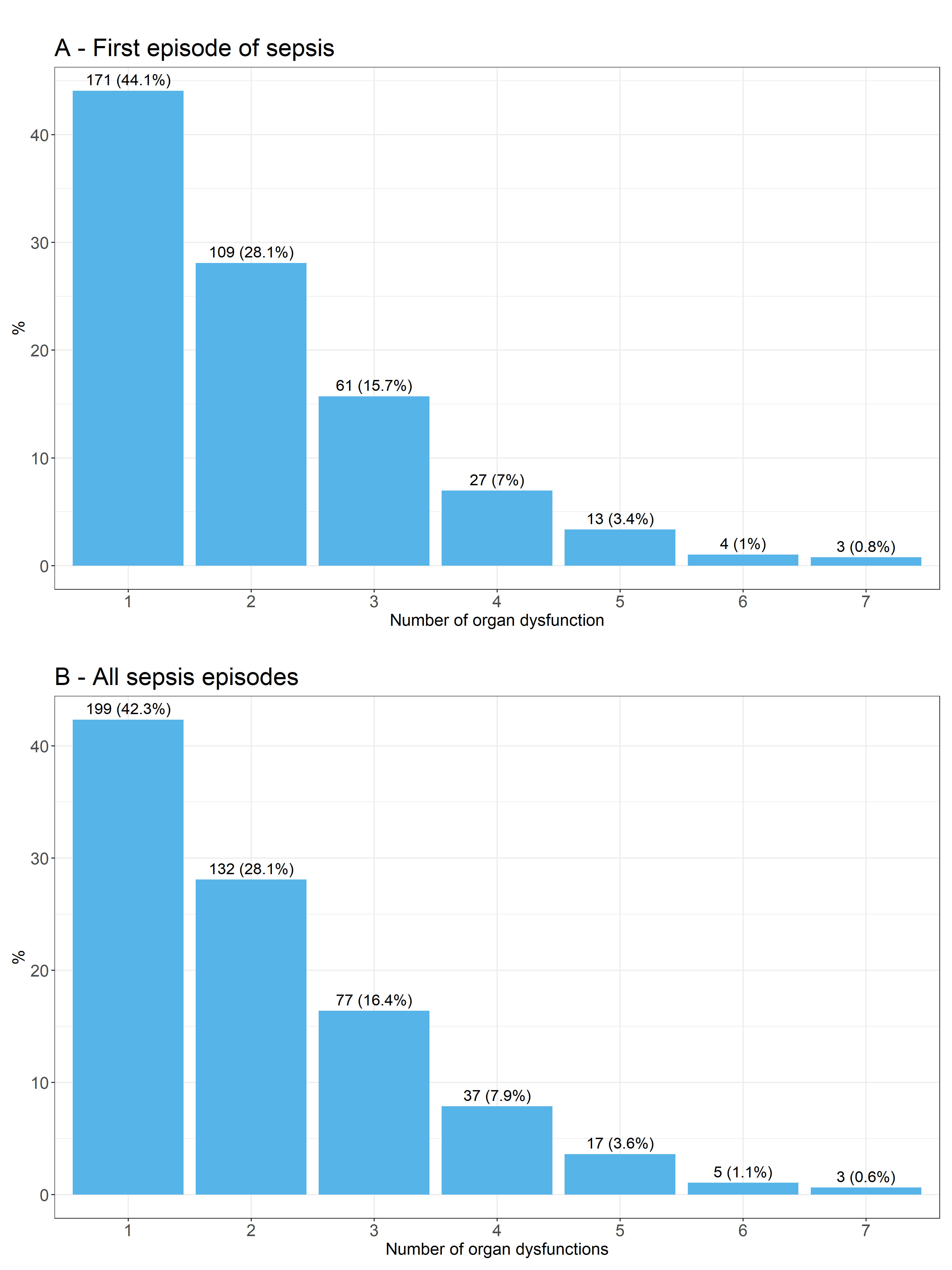


# **eFigure 5** – Patient locale at sepsis diagnosis. Top: Stratified according to outcome; Bottom: All patients.

**
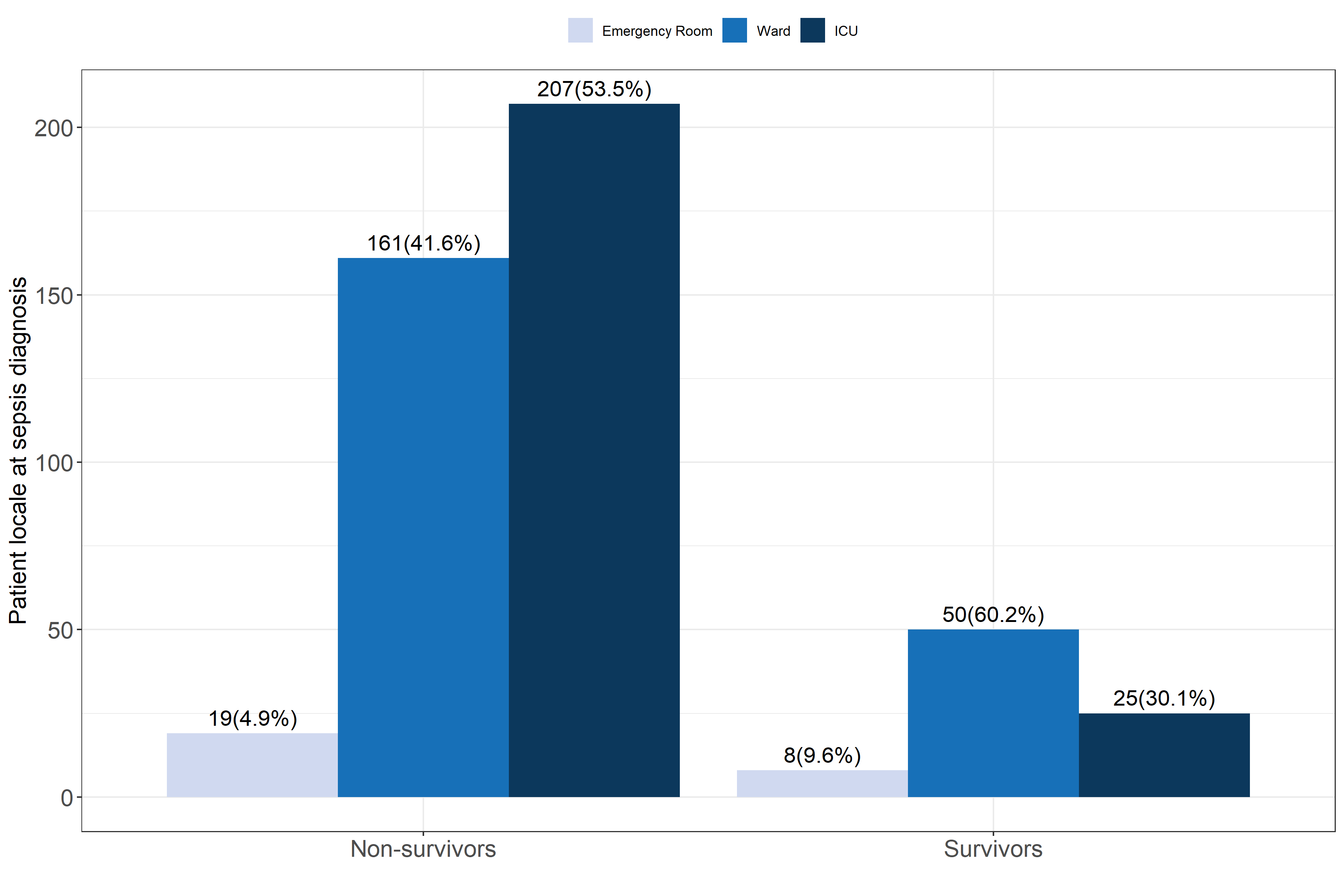
**
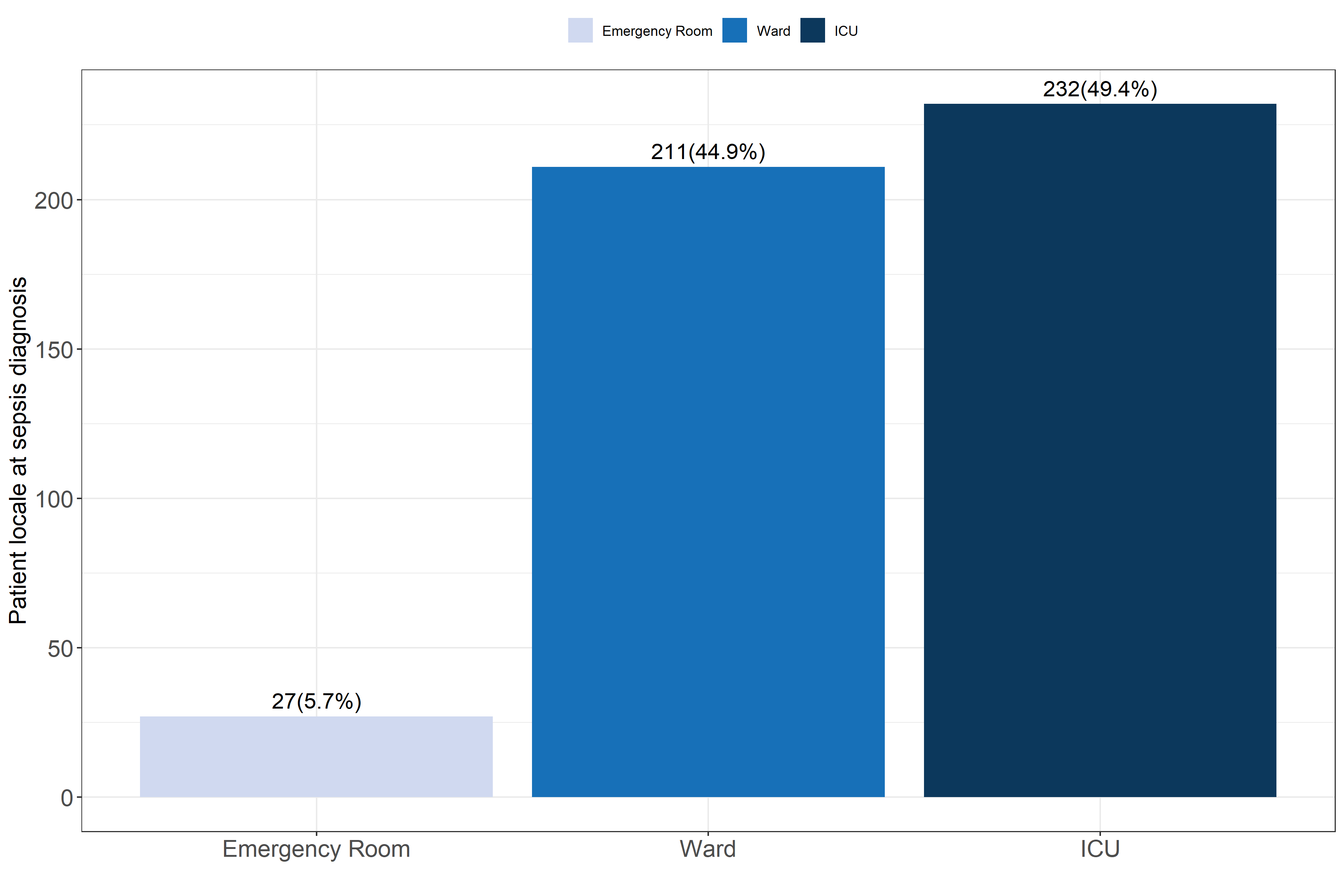


# **eTable 4 –** Pathogens isolated from cultures according to suspected site; more than one pathogen was possible for each patient. CRBI: Catheter related bloodstream infections. Note that pathogens could be isolated in any culture collected from the patient within the 48h timeframe. The local diagnosis was considered as reference; therefore the isolated pathogen could not be considered the culprit for the infection.

|  | Pneumonia | Unknown | Urinary Infection | Skin/Soft Tissues | CRBI | Other sources | Total |
| --- | --- | --- | --- | --- | --- | --- | --- |
| Escherichia coli | 12/197 (6.1%) | 9/82 (11%) | 49/143 (34.3%) | 2/21 (9.5%) | 2/28 (7.1%) | 8/102 (7.8%) | 82/573 (14.3%) |
| Outros Staphylococcus | 30/197 (15.2%) | 18/82 (22%) | 8/143 (5.6%) | 2/21 (9.5%) | 5/28 (17.9%) | 12/102 (11.8%) | 75/573 (13.1%) |
| Klebsiella pneumonia | 21/197 (10.7%) | 13/82 (15.9%) | 17/143 (11.9%) | 3/21 (14.3%) | 2/28 (7.1%) | 16/102 (15.7%) | 72/573 (12.6%) |
| Pseudomonas aeruginosa | 27/197 (13.7%) | 9/82 (11%) | 6/143 (4.2%) | 2/21 (9.5%) | 3/28 (10.7%) | 7/102 (6.9%) | 54/573 (9.4%) |
| Other gram-negative rods | 25/197 (12.7%) | 3/82 (3.7%) | 5/143 (3.5%) | 2/21 (9.5%) | 3/28 (10.7%) | 7/102 (6.9%) | 45/573 (7.9%) |
| Staphylococcus aureus | 12/197 (6.1%) | 5/82 (6.1%) | 4/143 (2.8%) | 1/21 (4.8%) | 6/28 (21.4%) | 8/102 (7.8%) | 36/573 (6.3%) |
| Candida albicans | 9/197 (4.6%) | 3/82 (3.7%) | 8/143 (5.6%) | 3/21 (14.3%) | 1/28 (3.6%) | 7/102 (6.9%) | 31/573 (5.4%) |
| Enterococcus faecalis | 2/197 (1%) | 2/82 (2.4%) | 10/143 (7%) | 0/21 (0%) | 1/28 (3.6%) | 5/102 (4.9%) | 20/573 (3.5%) |
| Acinetobacter baumannii | 7/197 (3.6%) | 1/82 (1.2%) | 4/143 (2.8%) | 2/21 (9.5%) | 2/28 (7.1%) | 1/102 (1%) | 17/573 (3%) |
| Candida sp | 4/197 (2%) | 6/82 (7.3%) | 2/143 (1.4%) | 1/21 (4.8%) | 0/28 (0%) | 2/102 (2%) | 15/573 (2.6%) |
| Candida glabrata | 2/197 (1%) | 2/82 (2.4%) | 4/143 (2.8%) | 0/21 (0%) | 1/28 (3.6%) | 5/102 (4.9%) | 14/573 (2.4%) |
| Other gram-positive coccus | 7/197 (3.6%) | 4/82 (4.9%) | 1/143 (0.7%) | 0/21 (0%) | 0/28 (0%) | 2/102 (2%) | 14/573 (2.4%) |
| Others | 5/197 (2.5%) | 0/82 (0%) | 4/143 (2.8%) | 0/21 (0%) | 0/28 (0%) | 5/102 (4.9%) | 14/573 (2.4%) |
| Klebsiella sp | 4/197 (2%) | 0/82 (0%) | 3/143 (2.1%) | 0/21 (0%) | 0/28 (0%) | 4/102 (3.9%) | 11/573 (1.9%) |
| Streptococcus, unspecified | 3/197 (1.5%) | 1/82 (1.2%) | 1/143 (0.7%) | 3/21 (14.3%) | 1/28 (3.6%) | 2/102 (2%) | 11/573 (1.9%) |
| Enterococcus faecium | 3/197 (1.5%) | 1/82 (1.2%) | 4/143 (2.8%) | 0/21 (0%) | 0/28 (0%) | 2/102 (2%) | 10/573 (1.7%) |
| Gram negative coccus | 4/197 (2%) | 0/82 (0%) | 4/143 (2.8%) | 0/21 (0%) | 0/28 (0%) | 1/102 (1%) | 9/573 (1.6%) |
| Enterococcus, unspecified | 5/197 (2.5%) | 0/82 (0%) | 2/143 (1.4%) | 0/21 (0%) | 1/28 (3.6%) | 1/102 (1%) | 9/573 (1.6%) |
| Streptococcus pneumoniae | 5/197 (2.5%) | 1/82 (1.2%) | 3/143 (2.1%) | 0/21 (0%) | 0/28 (0%) | 0/102 (0%) | 9/573 (1.6%) |
| Klebsiella oxytoca | 2/197 (1%) | 1/82 (1.2%) | 1/143 (0.7%) | 0/21 (0%) | 0/28 (0%) | 3/102 (2.9%) | 7/573 (1.2%) |
| Acinetobacter sp | 3/197 (1.5%) | 1/82 (1.2%) | 0/143 (0%) | 0/21 (0%) | 0/28 (0%) | 1/102 (1%) | 5/573 (0.9%) |
| Other gram-positive rods | 3/197 (1.5%) | 0/82 (0%) | 0/143 (0%) | 0/21 (0%) | 0/28 (0%) | 1/102 (1%) | 4/573 (0.7%) |
| Other yeasts | 2/197 (1%) | 0/82 (0%) | 0/143 (0%) | 0/21 (0%) | 0/28 (0%) | 1/102 (1%) | 3/573 (0.5%) |
| Other fungii | 0/197 (0%) | 1/82 (1.2%) | 1/143 (0.7%) | 0/21 (0%) | 0/28 (0%) | 1/102 (1%) | 3/573 (0.5%) |
| Clostridium, unspecified | 0/197 (0%) | 1/82 (1.2%) | 1/143 (0.7%) | 0/21 (0%) | 0/28 (0%) | 0/102 (0%) | 2/573 (0.3%) |
| Candida kruzei | 0/197 (0%) | 0/82 (0%) | 1/143 (0.7%) | 0/21 (0%) | 0/28 (0%) | 0/102 (0%) | 1/573 (0.2%) |

# **eTable 5 –** Positive cultures for patients that had one septic episode according to main definition. CRBI: Catheter related bloodstream infections. Note that pathogens could be isolated in any culture collected from the patient within the 48h timeframe. Same as in eTable 3, the final diagnosis was made by the site.

|  | Unknown | Urinary Infection | Skin/Soft Tissues | CRBI | Other sources | Total | Pneumonia |
| --- | --- | --- | --- | --- | --- | --- | --- |
| Escherichia coli | 2/28 (7.1%) | 2/19 (10.5%) | 4/17 (23.5%) | 0/5 (0%) | 0/3 (0%) | 4/10 (40%) | 12/74 (16.2%) |
| Outros Staphylococcus | 6/28 (21.4%) | 5/19 (26.3%) | 1/17 (5.9%) | 1/5 (20%) | 2/3 (66.7%) | 1/10 (10%) | 15/74 (20.3%) |
| Klebsiella pneumonia | 7/28 (25%) | 3/19 (15.8%) | 2/17 (11.8%) | 1/5 (20%) | 1/3 (33.3%) | 1/10 (10%) | 15/74 (20.3%) |
| Pseudomonas aeruginosa | 4/28 (14.3%) | 4/19 (21.1%) | 2/17 (11.8%) | 1/5 (20%) | 1/3 (33.3%) | 0/10 (0%) | 11/74 (14.9%) |
| Other gram-negative rods | 5/28 (17.9%) | 0/19 (0%) | 0/17 (0%) | 0/5 (0%) | 0/3 (0%) | 0/10 (0%) | 5/74 (6.8%) |
| Staphylococcus aureus | 5/28 (17.9%) | 2/19 (10.5%) | 0/17 (0%) | 0/5 (0%) | 0/3 (0%) | 0/10 (0%) | 7/74 (9.5%) |
| Candida albicans | 1/28 (3.6%) | 0/19 (0%) | 0/17 (0%) | 1/5 (20%) | 0/3 (0%) | 0/10 (0%) | 2/74 (2.7%) |
| Enterococcus faecalis | 1/28 (3.6%) | 2/19 (10.5%) | 1/17 (5.9%) | 0/5 (0%) | 0/3 (0%) | 1/10 (10%) | 5/74 (6.8%) |
| Acinetobacter baumannii | 4/28 (14.3%) | 1/19 (5.3%) | 0/17 (0%) | 1/5 (20%) | 0/3 (0%) | 0/10 (0%) | 6/74 (8.1%) |
| Candida sp | 2/28 (7.1%) | 2/19 (10.5%) | 1/17 (5.9%) | 1/5 (20%) | 0/3 (0%) | 0/10 (0%) | 5/74 (6.8%) |
| Candida glabrata | 0/28 (0%) | 0/19 (0%) | 0/17 (0%) | 0/5 (0%) | 0/3 (0%) | 0/10 (0%) | 0/74 (0%) |
| Other gram-positive coccus | 0/28 (0%) | 2/19 (10.5%) | 1/17 (5.9%) | 0/5 (0%) | 0/3 (0%) | 0/10 (0%) | 3/74 (4.1%) |
| Others | 0/28 (0%) | 0/19 (0%) | 2/17 (11.8%) | 0/5 (0%) | 0/3 (0%) | 2/10 (20%) | 4/74 (5.4%) |
| Klebsiella sp | 0/28 (0%) | 0/19 (0%) | 0/17 (0%) | 0/5 (0%) | 0/3 (0%) | 0/10 (0%) | 0/74 (0%) |
| Streptococcus, unspecified | 0/28 (0%) | 0/19 (0%) | 0/17 (0%) | 0/5 (0%) | 0/3 (0%) | 1/10 (10%) | 1/74 (1.4%) |
| Enterococcus faecium | 1/28 (3.6%) | 1/19 (5.3%) | 2/17 (11.8%) | 0/5 (0%) | 0/3 (0%) | 0/10 (0%) | 4/74 (5.4%) |
| Gram negative coccus | 1/28 (3.6%) | 0/19 (0%) | 1/17 (5.9%) | 0/5 (0%) | 0/3 (0%) | 0/10 (0%) | 2/74 (2.7%) |
| Enterococcus, unspecified | 0/28 (0%) | 0/19 (0%) | 0/17 (0%) | 0/5 (0%) | 0/3 (0%) | 1/10 (10%) | 1/74 (1.4%) |
| Streptococcus pneumoniae | 1/28 (3.6%) | 0/19 (0%) | 0/17 (0%) | 0/5 (0%) | 0/3 (0%) | 0/10 (0%) | 1/74 (1.4%) |
| Klebsiella oxytoca | 0/28 (0%) | 1/19 (5.3%) | 0/17 (0%) | 0/5 (0%) | 0/3 (0%) | 0/10 (0%) | 1/74 (1.4%) |
| Acinetobacter sp | 1/28 (3.6%) | 0/19 (0%) | 0/17 (0%) | 0/5 (0%) | 0/3 (0%) | 0/10 (0%) | 1/74 (1.4%) |
| Other gram-positive rods | 1/28 (3.6%) | 0/19 (0%) | 0/17 (0%) | 0/5 (0%) | 0/3 (0%) | 0/10 (0%) | 1/74 (1.4%) |
| Other yeasts | 1/28 (3.6%) | 0/19 (0%) | 0/17 (0%) | 0/5 (0%) | 0/3 (0%) | 1/10 (10%) | 2/74 (2.7%) |
| Other fungii | 0/28 (0%) | 1/19 (5.3%) | 1/17 (5.9%) | 0/5 (0%) | 0/3 (0%) | 0/10 (0%) | 2/74 (2.7%) |
| Clostridium, unspecified | 0/28 (0%) | 0/19 (0%) | 0/17 (0%) | 0/5 (0%) | 0/3 (0%) | 0/10 (0%) | 0/74 (0%) |
| Candida kruzei | 0/28 (0%) | 0/19 (0%) | 1/17 (5.9%) | 0/5 (0%) | 0/3 (0%) | 0/10 (0%) | 1/74 (1.4%) |

# **eFigure 6** – (A) Infection source considering first alternative definition. The number of patients that did not receive antibiotic reflects patients that developed new organ failure in the absence of any other clinically relevant event and were considered as possibly septic under this definition. (B) Organ dysfunction at presentation for the alternative definition 1 analysis.


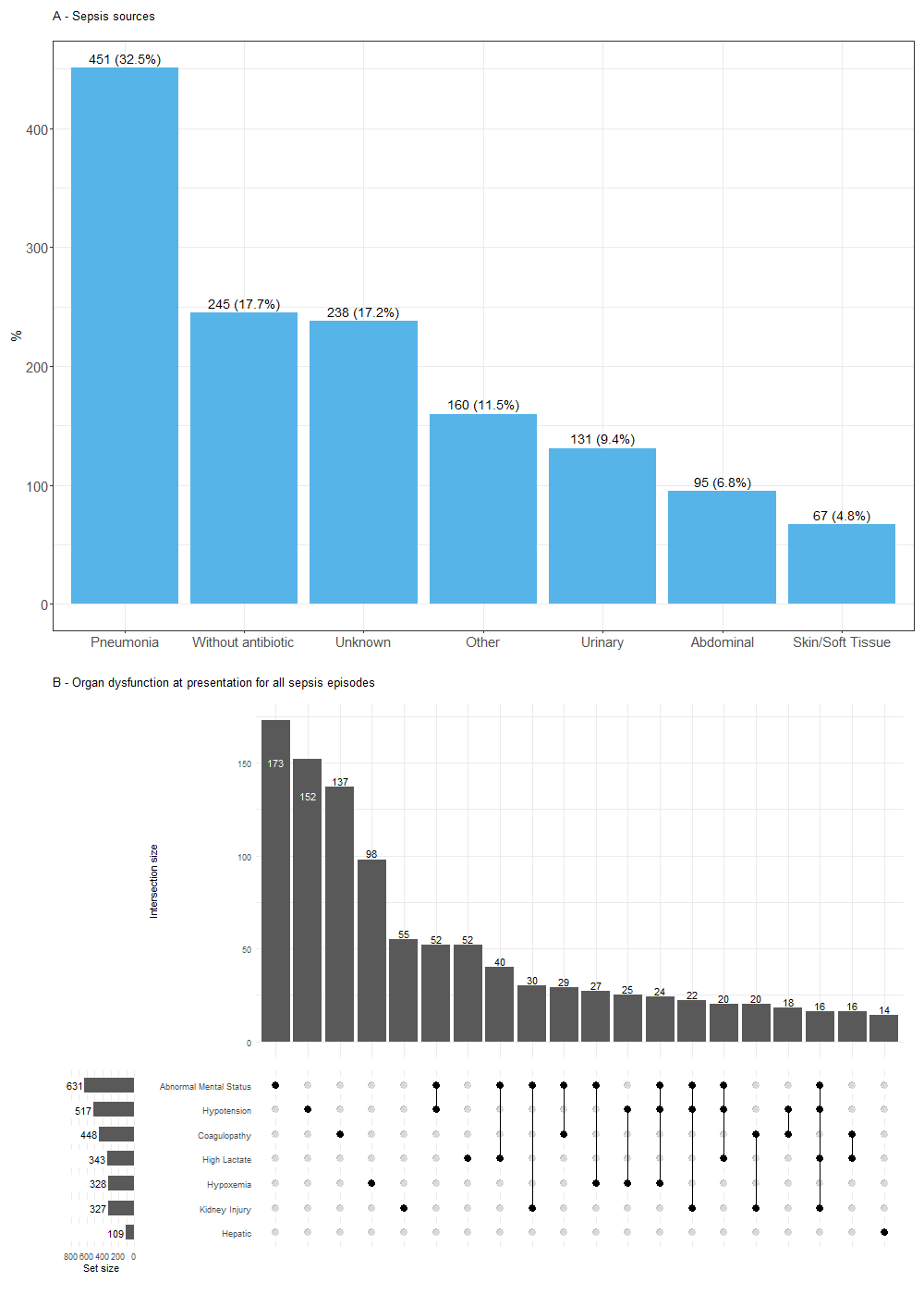


# **eFigure 7** – Distribution of odds ratio (upper row) and AF (lower row) according to admission type (columns) for the first alternative definition considering the effects of definitive, very probably, and probable sepsis.


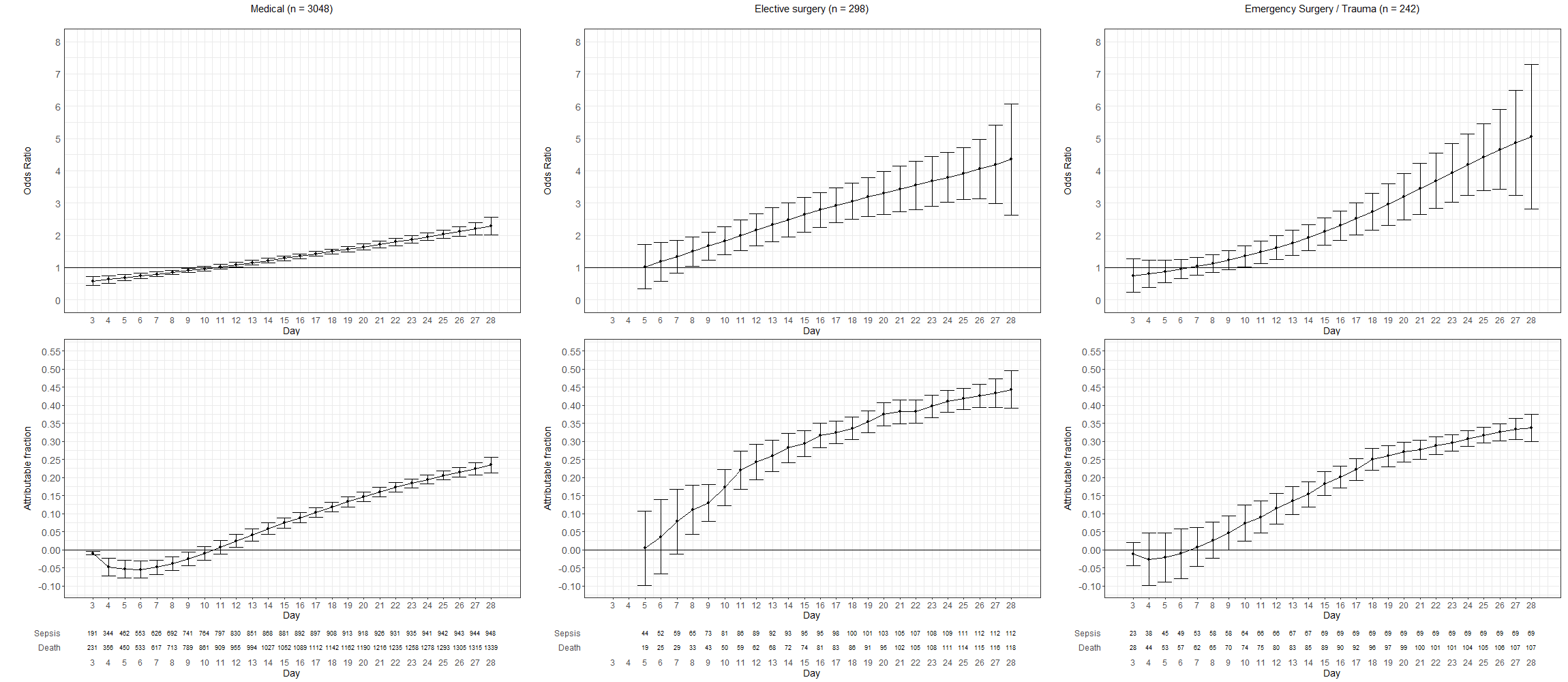


# **eFigure 8** – Sepsis sources (A) and organ dysfunction at presentation for the second alternative definition analysis.

**
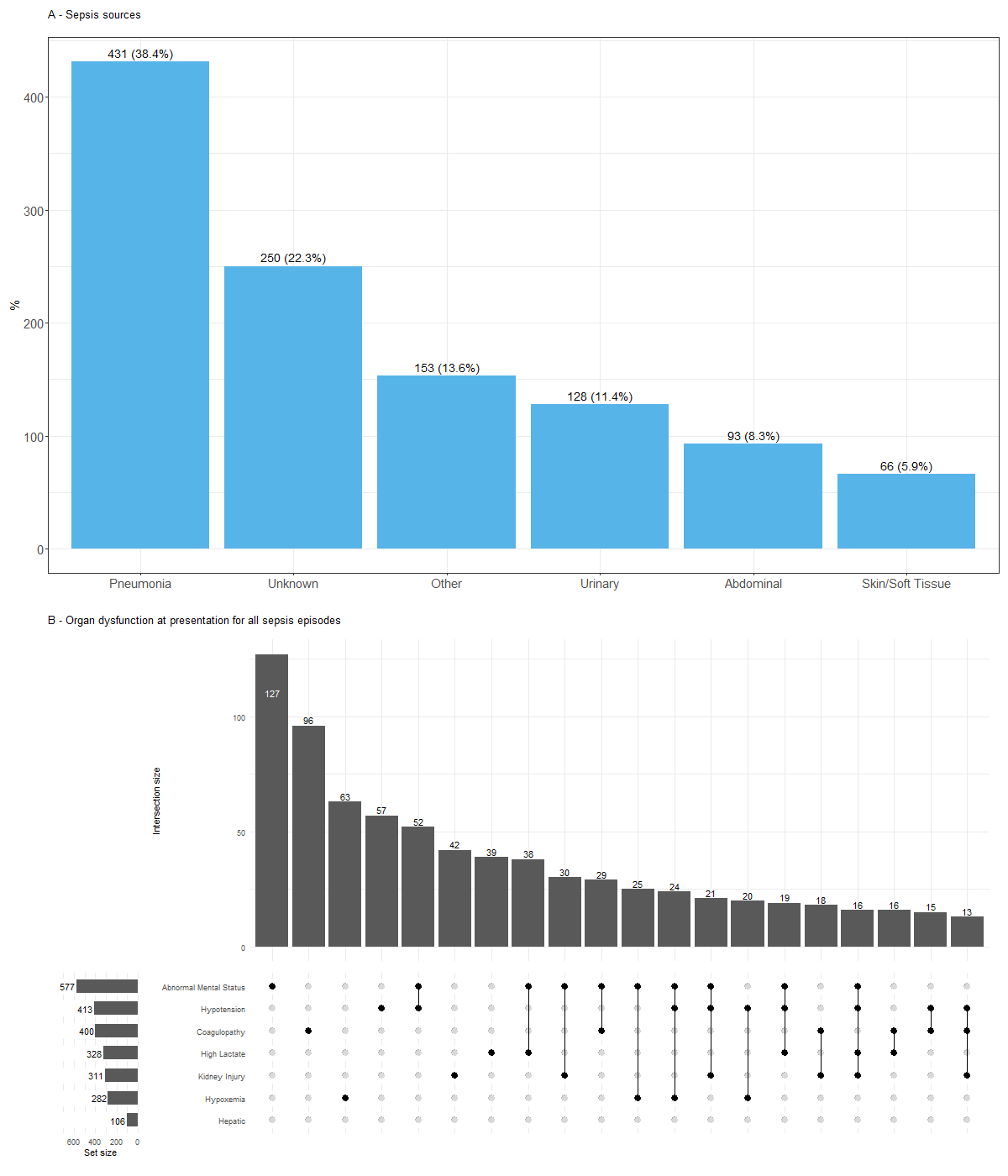
**

# **eFigure 9** – Distribution of odds ratio (upper row) and PAF (lower row) according to admission type (columns) for the second alternative definition considering the effects of definitive and very probably sepsis.


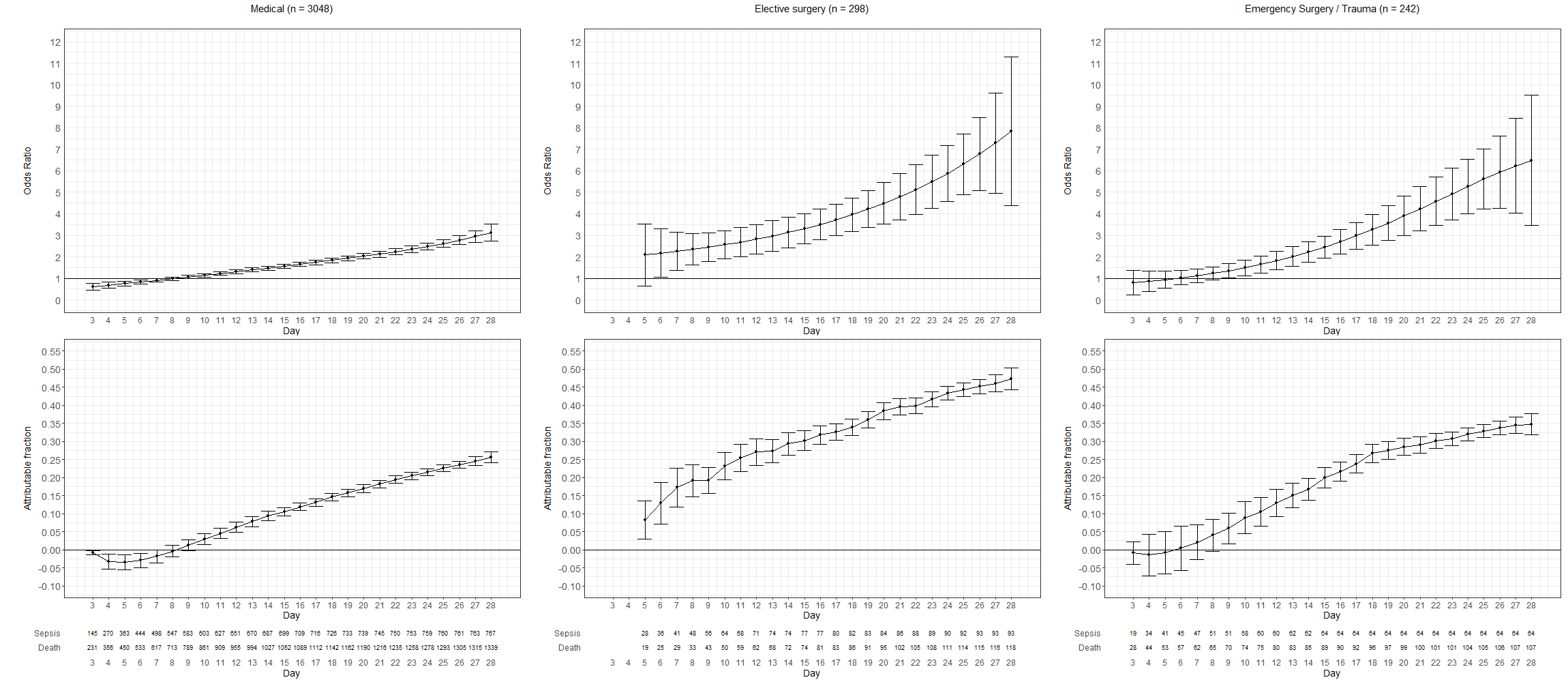


# **eFigure 10 -** Distribution of odds ratio (upper row) and PAF (lower row) according to admission type (columns) for the post-hoc definition based on SOFA score


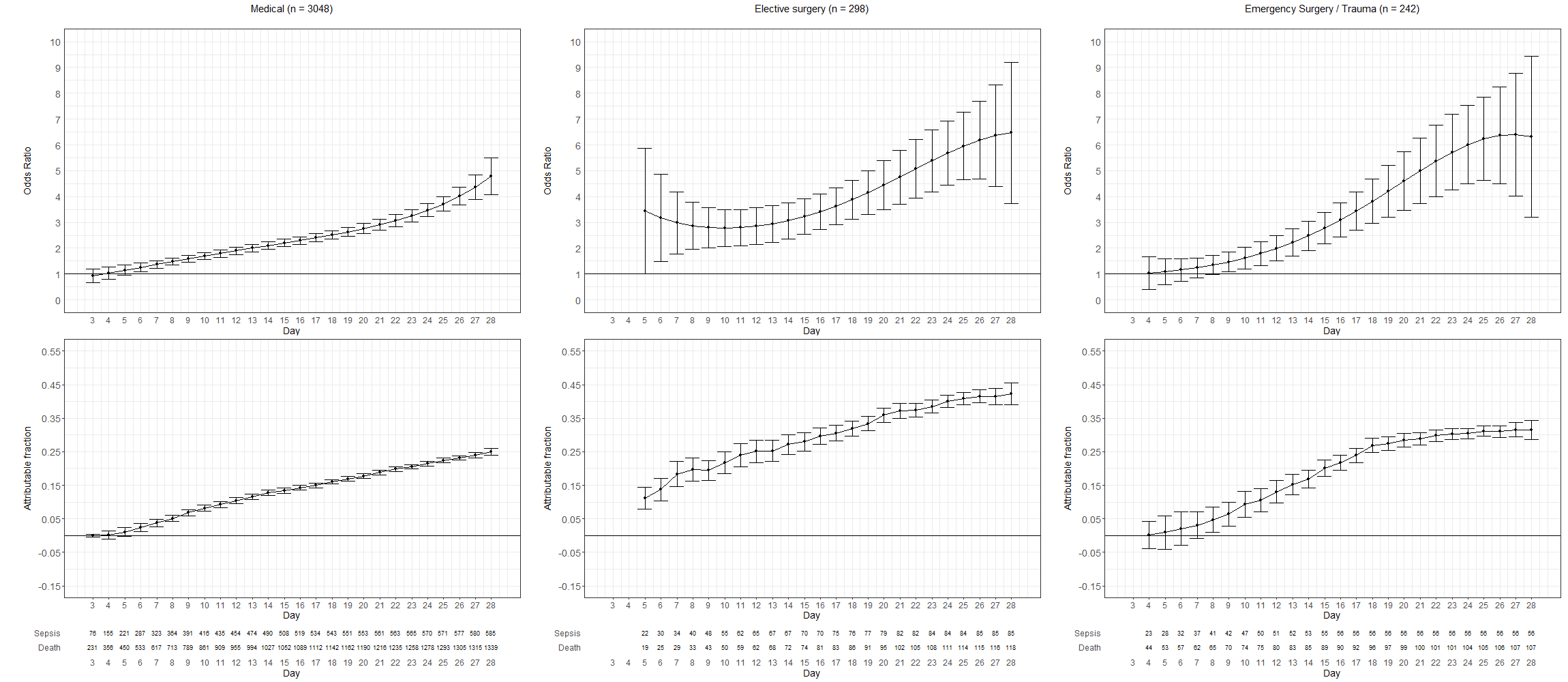


# **eFigure 11 -** Distribution of odds ratio (upper row) and PAF (lower row) according to admission type (columns) after excluding patients with infection at baseline.


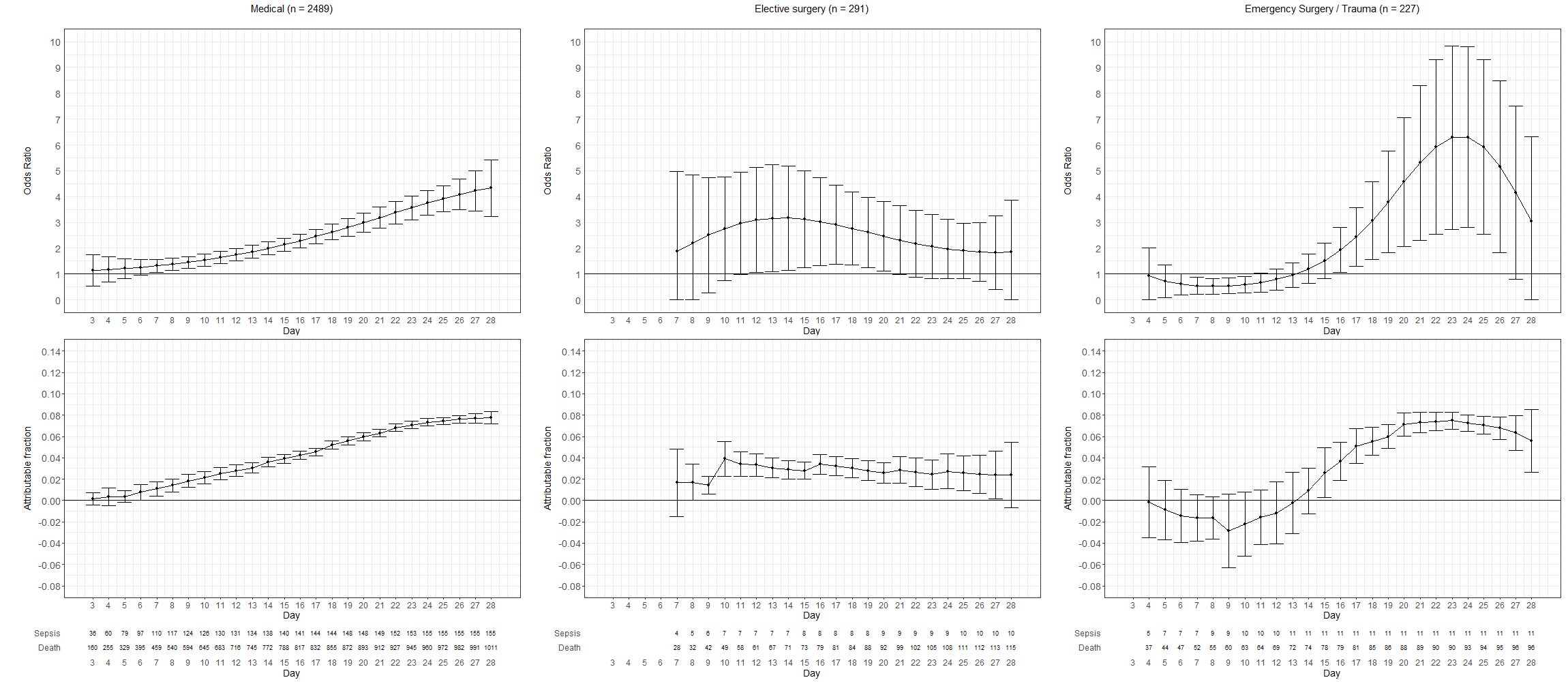


# **eFigure 12 –** Distribution of odds ratio (upper row) and PAF (lower row) for nosocomial sepsis on medical admissions using time-dependent admission type as additional adjustment in the model


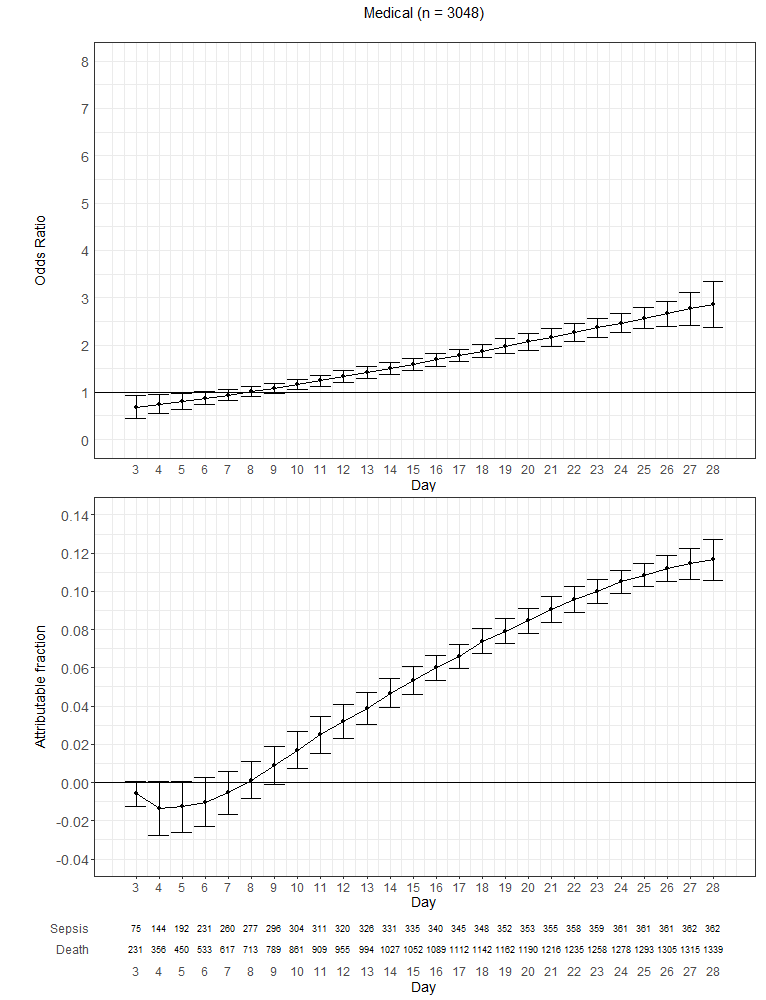


# **eFigure 13 –** Distribution of odds ratio (upper row) and PAF (lower row) of admission types for medical admission using time-dependent admission type as additional adjustment in the model for the main definition. Note how infection reasons for admission have the higher AF, mostly stable during hospitalization


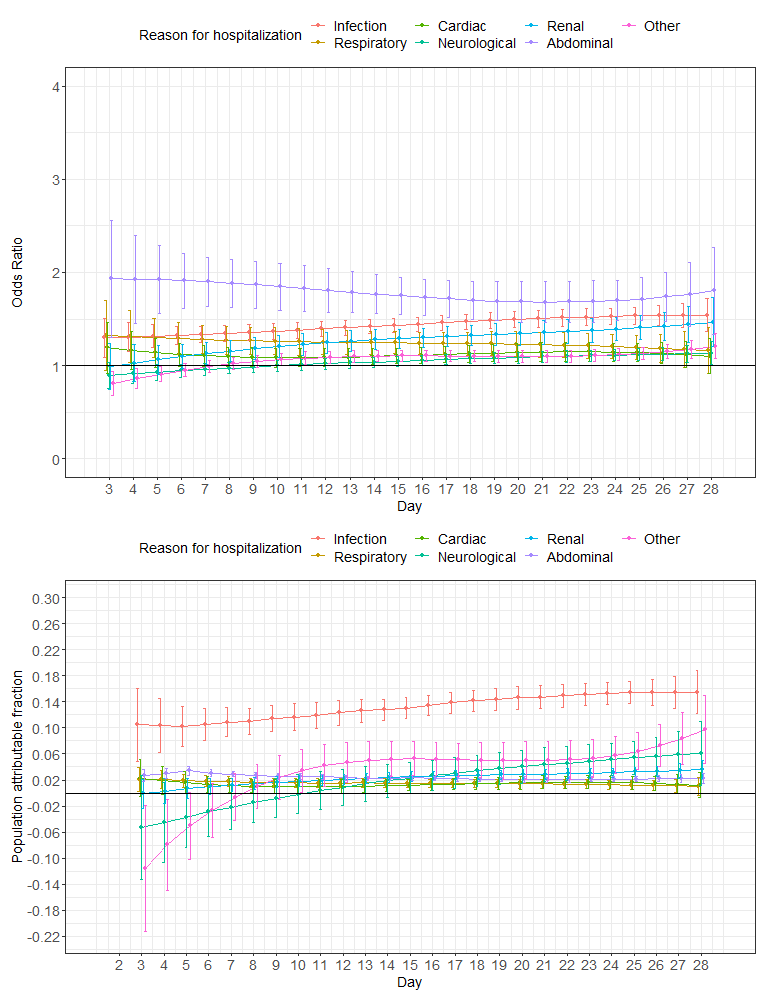


# **eFigure 14 -** Comparison of the three definitions for elective surgery admissions. Upper panel: The daily odds ratio for mortality in elective surgery patients according to the definition used. Bottom panel: The respective AF for each definition. Note that a higher OR does not equal higher AF since prevalence of events also changes.

**
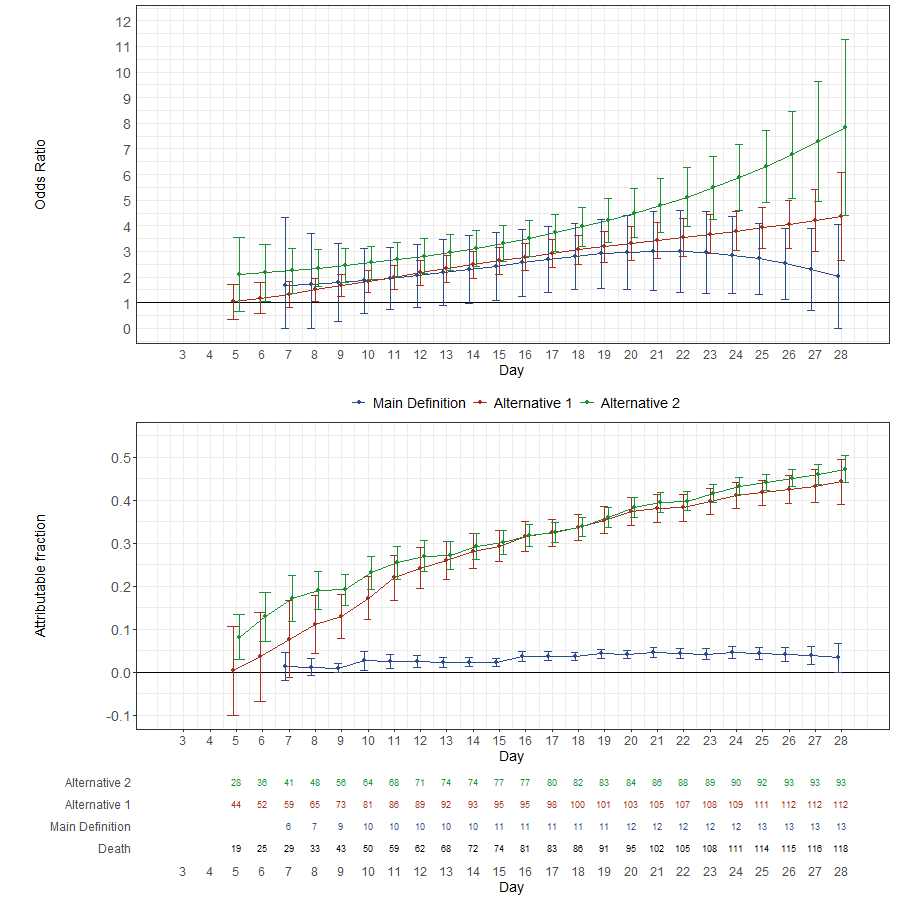
**

# **eFigure 15** – Comparison of the three definitions for emergency surgery admissions. Upper panel: The daily odds ratio for mortality in medical patients according to the definition used. Bottom panel: The respective AF for each definition. Note that a higher OR does not equal higher AF since prevalence of events also changes.

**
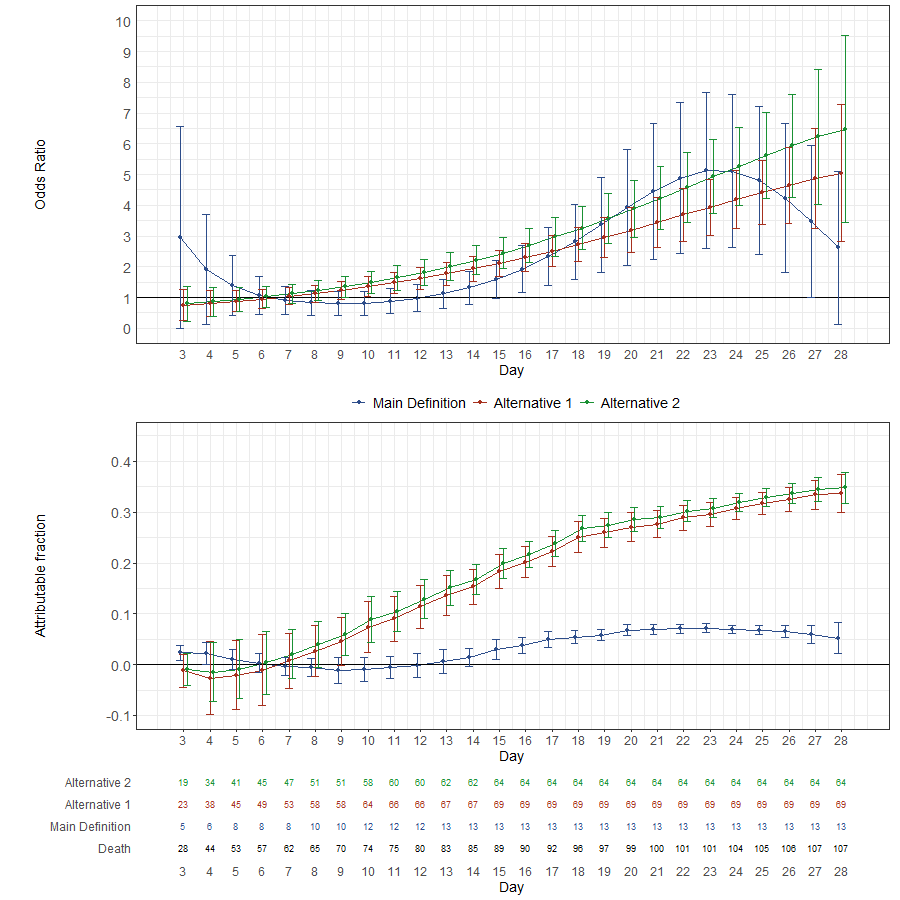
**

# **eTable 6 –** Comparison of estimates of the OR for nosocomial sepsis for the main definition and the Sepsis 3 definitions without adjustment for admission type (adjustment to infection at admission only) and with adjustment for admission type. Note that estimates are similar.

| **Definition and Analysis** | **Average OR of death due to sepsis** | **Average attributable fraction for death of sepsis** |
| --- | --- | --- |
| **Main Definition** |  |  |
| Primary analysis | **1.73 (95% CI 1.60; 1.87)** | **0.076 (95% CI 0.068; 0.084)** |
| Adding admission type to adjustment | **1.64 (95% CI 1.51; 1.76)** | **0.070 (95% CI of 0.061; 0.0782)** |
| **Sepsis 3 (increase SOFA) definition** |  |  |
| Primary adjustment | **2.25 (95% CI 2.10; 2.39)** | **0.176 (95% CI 0.167; 0.185)** |
| Adding admission type to adjustment | **2.39 (95% CI 2.24; 2.54)** | **0.184 (95% CI 0.176; 0.192)** |
