# Supplementary material for: Attributable mortality due to nosocomial sepsis in Brazilian hospitals: a case–control study
Source: Ann Intensive Care. 2023 Apr 26;13:32. doi: 10.1186/s13613-023-01123-y (PMC10133434; doi:10.1186/s13613-023-01123-y)
Supplement: Supplementary file 2 — Additional file 2: IMPACTO-MAPA study. [file 13613_2023_1123_MOESM2_ESM.pdf]

MAPA STUDY

PROTOCOL PDF

1. JULY 2018
2. APRIL 2019

IMPACTO-MAPA STUDY

Identifying the Mortality Attributable to Sepsis in Hospitalized PAtients in Brazil: IMPACTO-MAPA

A study of the Platform of projects in support of the National Plan of Action for the Prevention and Control of Antimicrobial Resistance – IMPACTO MR Program

National Coordinator: Institute of Research of the *Hospital do Coração* (IP-HCor)
Rua Abílio Soares, 250 – Paraíso
CEP: 04005-000 São Paulo, SP – Brazil
Phone: ++55 11 3053 6611 Ext: 8210
Fax: ++55 11 3886 4695

Sponsor: Institute of Education and Research of the *Hospital do Coração* (IP-HCor)

Steering Committee: Fernando Godinho Zampieri
Alexandre Biasi Cavalcanti

THE CONTENT OF THIS RESEARCH PROTOCOL IS THE INTELLECTUAL PROPERTY OF IP-HCOR. THE REPRODUCTION OR USE OF THE INFORMATION AND DATA CONTAINED HEREIN IS NOT ALLOWED FOR ANY PURPOSE OTHER THAN THAT PROPOSED BY THE STUDY WITHOUT THE PRIOR FORMAL AUTHORIZATION OF THE AUTHORS.

Version 1.0 of November 26, 2010.

STUDY FLOWCHART

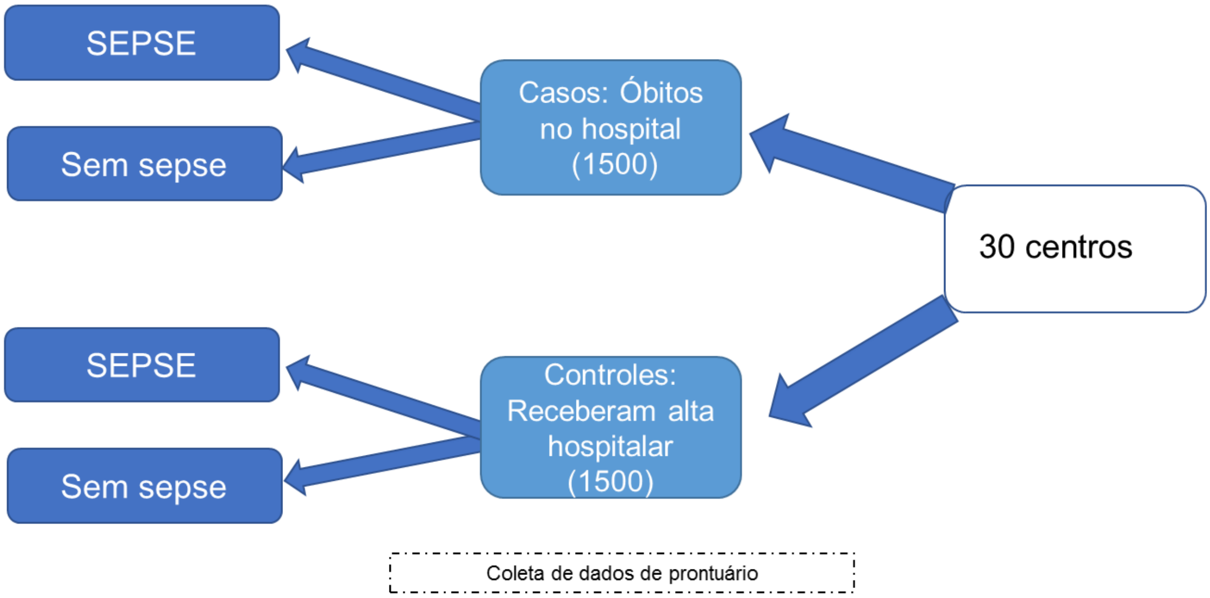

Caption:
SEPSE = SEPIS
Sem SEPSE = No SEPIS
Casos: Óbitos no hospital (1500) = Cases: Deaths in the hospital (1500)
Controles: Receberam alta hospitalar (1500) = Controls: Patients discharged (1500)
30 centros = 30 centers
Coleta de dados de prontuário = Collection of medical record data

**Title**	**IMPACTO-MAPA Study**<br>**Identifying the Mortality Attributable to Sepsis in Hospitalized Patients in Brazil**<br>**A study of the Platform of projects in support of the National Plan of Action for the Prevention and Control of Antimicrobial Resistance – IMPACTO MR Program**
**Coordinating Center**	Institute of Research of the *Hospital do Coração* (IP-HCor)<br>Rua Abílio Soares, 250 – Paraíso<br>CEP: 04005-000 São Paulo, SP – Brazil<br>Phone: ++55 11 3053 6611 Ext: 8210<br>Fax: ++55 11 3886 4695
**Study Design**	Observational case-control study
**Primary objective**	To measure the mortality attributable to sepsis in hospitalized patients in Brazil.
**Secondary objectives**	1. To evaluate the length of hospital stay in patients with or without sepsis<br>2. To evaluate the mortality attributable to other hospitalization complications (infarction, cerebrovascular accident, pulmonary thromboembolism)<br>3. To evaluate the completeness quality of the medical record data<br>4. To evaluate the quality and consistency of the filling of the declaration of hospital deaths
**Eligibility of cases and controls**	Each hospital will provide clinical, demographic, and laboratory data for the last 50 deaths at the institution. The controls will be the data of the patient who was discharged alive with the closest date and time to the date of death of the case.
**Sample size**	30 hospitals, 100 patients in each hospital (50 cases and 50 controls), amounting to 3,000 patients

SUMMARY

INTRODUCTION.....5
OBJECTIVES OF THE STUDY.....7
METHODS.....7
STUDY DESIGN.....7
SAMPLE SIZE.....7
DATA COLLECTION.....8
STATISTICAL ANALYSIS.....11
ADDITIONAL OBJECTIVES:.....13
DATA MANAGEMENT.....13
STUDY SCHEDULE.....14
DISSEMINATION OF RESULTS.....15
PROJECT RISKS.....15
ETHICAL CONSIDERATIONS.....16
REFERENCES.....16

Introduction

Hospital admission is not free from risk. Admitted patients are subject to different clinical complications that may represent important health problems, leading to an increase in morbidity and mortality. These complications are also associated with increased costs, which are caused not only by increased hospitalization time but also by the need for additional procedures and treatments, in addition to the patient's greater absence from their daily life. Thus, it is important to understand the frequency and contribution of clinical complications in hospitalized patients.

Among the most frequent complications, we can highlight the occurrence of nosocomial infection. In developing countries, nosocomial infections are extremely frequent and affect more than 15 out of every 100 hospitalized patients [1]. In some more severe situations, the infection causes the dysfunction of one or more organs, a situation called sepsis and which has high morbidity and mortality [2]. Despite its frequency, the mortality attributable to sepsis, that is, the increase in mortality resulting from the occurrence of nosocomial sepsis in a patient is unknown.

There is a great amount of data on the prevalence and incidence of sepsis and the mortality of patients with sepsis, including in Brazil [3]. We

also have some data on the mortality attributable to sepsis among patients with sepsis, that is, the total number of deaths that can be attributed directly to sepsis rather than other concomitant problems. For example, in patients admitted to the ICU, the mortality fraction attributable to sepsis is approximately 15% in developed countries [4]. However, from the point of view of health management, it is fundamental to understand what proportion of total deaths in the population can be attributed to sepsis. This information is obviously critical for decisions on the allocation of investment in care, training and research. However, there is no good information on the proportion of deaths that is attributable to sepsis, not even from other countries. Our proposal is to estimate, among the cases of hospital deaths, which proportion is attributable to sepsis versus other groups of causes (cardiovascular diseases, cancer, etc.).

The understanding of the mortality attributable to sepsis has several relevant operational and academic implications. From the operational point of view, the measurement of the attributable fraction of mortality allows estimating the impact of future interventions aimed at reducing the occurrence of sepsis (hand hygiene campaign, Hospital Infection Commission efforts, among others). From the academic point of view, this information will allow a better calculation of the sample size for future clinical trials in the area.

Objectives of the study

Primary: To measure the mortality attributable to sepsis in hospitalized patients in Brazil.

Secondary:

1. Length of hospital stay in patients with or without sepsis
2. To evaluate the mortality attributable to other hospitalization complications (infarction, cerebrovascular accident, pulmonary thromboembolism)
3. To evaluate the completeness quality of the medical record data
4. To evaluate the quality and consistency of the filling of the declaration of hospital deaths

Methods

Study design

Case-control study to be performed using medical record analysis.

Sample size

Sample calculation was performed using the Fleiss, Tytun, and Ury method to estimate the power of a two-tailed test for proportion difference using the Hmisc package of R software (version 3.5.0). Sample size was estimated for

a power above 90% for an odds ratio of at least 1.4 assuming an incidence of nosocomial sepsis of 15% in the controls. Thus, 2,700 patients would be needed. We chose to round the number to 3,000 so as to ensure a margin of safety. This total of patients will be included in a group of 30 Brazilian hospitals that fulfill the following characteristics:

1. At least 100 active beds
2. Presence of intensive care unit and emergency room
3. Availability to collect data for the study

Each hospital will provide clinical, demographic, and laboratory data for the last 50 deaths at the institution. The controls will be the data of the patient who was discharged alive with the closest date and time to the date of death of the case. Fifty controls per hospital will be used, amounting to 100 patients per hospital. The consecutive pairing with the closest living discharge will ensure the minimization of any temporal impacts.

Data collection

Data collection will be performed by each hospital using an electronic data collection system designed specifically for the study. The filling in will be done by each center based on medical record data, according to local availability, respecting the maximum term of two years. All data collected will be anonymous, in order to preserve the identity of the participants and to ensure confidentiality. The data to be collected are:

1. Demography: Sex, age, education level, comorbidities (Charlson Comorbidity Index)
2. Reason for hospital admission: Scheduled versus unscheduled. Presence of infection as a cause of hospital admission (defined as hospitalization with primary diagnosis of infectious disease by ICD-10).
3. Physiological variables: Blood pressure, heart rate, respiratory rate, level of consciousness.
4. Laboratory variables: Renal function (serum creatinine), blood count, C-reactive protein (if available), coagulogram, bilirubin, serum lactate.
5. Outcome of all cultures (blood, tracheal secretion, urine, or other sites) collected during hospitalization.
6. Length of hospital stay
7. Use of intrahospital resources
    a. Number of imaging tests performed to elucidate the focus of infection
    b. Need for surgical procedures
    c. Intensive care stay
    d. Use of supportive therapy such as mechanical ventilation, renal replacement therapy, use of vasopressors.

Laboratory and physiological variables will be collected from the admission and during the first three days of the patient's hospitalization. When an antimicrobial treatment is added, started, or changed and/or some type of microbiological examination (culture) is performed, we will again

collect physiological and laboratory data for an additional period of three days before and after the date of the occurrence to follow the occurrence of organ dysfunction associated with suspected infection (sepsis). In these situations, we will also note:

1. The cause of sepsis
2. The prescribed antibiotic and its suitability (i.e. whether the antibiotic prescribed at the initial time was effective against the pathogen that eventually proved to be the cause of the event)
3. Whether or not the infection was associated with a surgical procedure or invasive device (prosthesis infection, catheter-related bloodstream infection, etc.).

We will also note the occurrence of other clinical complications, such as:

1. Pulmonary thromboembolism
2. Myocardial ischemia/Myocardial infarction
3. Cerebrovascular accident (ischemic or hemorrhagic)

For the cases (that is, for hospital deaths), we will collect the information present in the death certificate (immediate cause and its sequence, as well as contributing causes).

Definition of the exposure factor: The exposure of interest is the nosocomial sepsis, that is, nosocomial infection that occurs with the occurrence of organ dysfunction. As organ dysfunction, we will consider the presence of at least

one of the following factors when the therapeutic antimicrobial treatment is started or changed in hospitalized patients:

1. Systolic pressure < 90 mmHg or mean pressure < 65 mmHg or drop in pressure > 40 mmHg
2. PaO2/FiO2 ratio <300 or need for O2 to keep SpO2 > 90
3. Decreased level of consciousness
4. Creatinine > 2.0 mg/dL or diuresis below 0.5mL/kg/h in the last 2 hours
5. Bilirubin > 2mg/dL
6. Platelet count < 100,000mm3
7. Lactate above reference value
8. Coagulopathy (INR > 1.5 or APTT > 60 sec)

Statistical analysis

The traditional univariate analysis comparing the groups (case and control) related to the variables described above will be performed using t-test or Mann-Whitney U test for continuous variables (as appropriate depending on the occurrence of normality) and Chi square test for comparison of proportions.

The main analysis (attributable mortality) will be done using logistic regression with predictors of event (hospital death) as predictors. Predictors will be chosen based on their clinical relevance, which will include:

1. Age
2. Type of admission

3. Comorbidities, as measured by Charlson comorbidity index
4. Occurrence of infection during hospitalization
5. Occurrence of multidrug-resistant infection, with emphasis on the presence of documented infection of carbapenem-resistant bacteria, including: *Acinetobacter baumannii, Pseudomonas aeruginosa, Serratia sp, Klebisiella pneumoniae*

Mortality attributable to sepsis will be calculated through the attributable fraction using the ratio of counterfactual probabilities (absence and presence of hospital sepsis) obtained by the logistic regression model.

In addition, we will evaluate the impact of the occurrence of nosocomial infection during the hospital stay through a competitive risk analysis (Finn & Gray), considering death as a competitor for the occurrence of an infectious event. With this model, we can estimate the fraction of additional hospitalization days attributable to nosocomial infection. The Finn & Gray model will correct the same predictors of traditional logistic regression.

We plan to carry out a marginal structural model (MSM) analysis that modulates the daily sequential data of the patient with outcomes. Through this approach, we can use the data collected on the days before and after the infection for more robust causality inference. The MSM design will be based on the behavior of the collected physiological and laboratory variables.

Some sensitivity analyses will be performed. We plan to analyze the mortality attributable to sepsis according to specific infectious foci

(bloodstream infection and nosocomial pneumonia) and according to the presence of infections by multidrug-resistant bacteria. We will also evaluate whether the use of appropriate antibiotics modulates the association between nosocomial sepsis and outcome.

Additional Objectives:

The study will provide regional data on the occurrence of complications in hospitalized patients; thus, a better targeting of resources for prevention may be carried out in the future. We plan to analyze the quality of the data included in patient records. The review of the medical records of 3,000 hospitalized patients will also allow us to point out the quality of the data completeness, eventual failures in the filing of death certificates, among others. This may also assist in developing campaigns to improve the clinical records of hospitalized patients.

Data management

All data will be included in a cloud-based electronic system developed specifically for the study. Measures to ensure the filling and quality of the data will be used, including:

1. Face-to-face and/or distance training for all researchers
2. Meeting of researchers
3. Remote monitoring of data filling
4. Frequent contact with centers (face-to-face and distance)
5. Audit and adjudication of the exposure of interest (sepsis) in all cases.

Study Schedule

The total duration of the study is three years, with the following schedule:

Year/Semester	Year 1/1	Year 1/2	Year 2/1	Year 2/2	Year 3/1	Year 3/2
**Project coordination**						
Project management	X	X	X	X	X	X
Quarterly meetings with technicians of the MH	X	X	X	X	X	X
Annual Meetings with Steering Committee	X	X	X	X	X	X
**Approval of centers**						
Initial submission of the protocol to the coordinating center	X	X	X	X	X	X
Follow-up of protocol submissions and approvals in the centers	X	X	X	X	X	X
Submission of partial and final reports to CEP	X	X	X	X	X	X
Payment of fee to CEP	X	X	X	X	X	X
**Management of centers (inclusion of patients)**						
Selection of centers	X	X				
Development and printing of support material	X					
Training of centers	X	X	X	X	X	X
Stimulation of patient inclusion	X	X	X	X	X	X
Assistance to researchers in conducting the protocol	X	X	X	X	X	X
Monitoring	X	X	X	X	X	X
Payment of researchers	X	X	X	X	X	X
**Data Management and analysis of results**						
CRF elaboration (printed and electronic)	X	X				X
System maintenance	X	X	X	X	X	X
Cleaning of data		X	X	X	X	X
Closing of the database					X	X
Data analysis						X
**Publication**						
Submission of results for publication/congress						X

Dissemination of Results

The Steering Committee of the IMPACTO MR-MAPA Study undertakes to publish its results, whatever they may be. Because it will be a large-scale randomized collaborative study, we aim to refer the main publications to high impact journals. The study data will be made available immediately to the Ministry of Health.

We intend to give access to the study database to other researchers. In the first two years after the publication of the main study, researchers will focus on sub-studies proposed by collaborative researchers. At this stage, the database will be kept under the custody of the study coordinators, and access will be allowed to third parties only upon express authorization of the Steering Committee of the IMPACTO MR-MAPA study after evaluation of the proposal accompanied by a statistical analysis plan.

Project Risks

We believe that the IMPACTO MR-MAPA study presents a low risk. We will perform a retrospective analysis using medical records in 30 Brazilian hospitals. Data collection will be done anonymously through a computerized central system. All analyses will be performed without exposing the patients' names or individualized information of the participating hospitals. The data collection system will be designed in a way that minimizes the input of incorrect information. The data inserted will be checked by the data management of the study.

Ethical considerations

This will be a case-control study, using a retrospective analysis of medical record data, with anonymous data collection. In this way, we anticipate that the risk for patients and institutions is minimal. Because of the very nature of the study, we will request exemption from obtaining the informed consent from the centers. There will be no contact or interaction with the included patients.

References

1. Allegranzi B, Bagheri Nejad S, Combescure C, Graafmans W, Attar H, Donaldson L, Pittet D. Burden of endemic health-care-associated infection in developing countries: systematic review and meta-analysis. Lancet. 2011 Jan 15;377(9761):228-41

2. Singer M, Deutschman CS, Seymour CW, Shankar-Hari M, Annane D, Bauer M, Bellomo R, Bernard GR, Chiche JD, Coopersmith CM, Hotchkiss RS, Levy MM, Marshall JC, Martin GS, Opal SM, Rubenfeld GD, van der Poll T, Vincent JL, Angus DC. The Third International Consensus Definitions for Sepsis and Septic Shock (Sepsis-3). JAMA. 2016 Feb 23;315(8):801-10

3. Machado FR, Cavalcanti AB, Bozza FA, Ferreira EM, Angotti Carrara FS, Sousa JL, Caixeta N, Salomao R, Angus DC, Pontes Azevedo LC; SPREAD Investigators; Latin American Sepsis Institute Network. The epidemiology of sepsis in Brazilian intensive care units (the Sepsis

PREvalence Assessment Database, SPREAD): an observational study. Lancet Infect Dis. 2017;17(11):1180-1189

4. Shankar-Hari M, Harrison DA, Rowan KM, Rubenfeld GD. Estimating attributable fraction of mortality from sepsis to inform clinical trials. J Crit Care. 2018 Jan 25;45:33-39

IMPACTO-MAPA STUDY

Identifying the Mortality Attributable to Sepsis in Hospitalized PAtients in

Brazil: IMPACTO-MAPA

A study of the Platform of projects in support of the National Plan of Action for the Prevention and Control of Antimicrobial Resistance – IMPACTO MR Program

National Coordinator:
Research Institute of Hospital do Coração (IP-HCor)
Rua Abilio Soares, 250 – Paraíso
CEP: 04005-000 São Paulo, SP – Brazil
Tel : 11 3053 6611 Extension: 8210
Fax: 11 3886 4695

Sponsor: Hcor Research Institute (IP-HCor)

Steering Committee: Fernando Godinho Zampieri
Alexandre Biasi Cavalcanti

THE CONTENT OF THIS RESEARCH PROTOCOL IS THE INTELLECTUAL PROPERTY OF IP-HCOR. THE REPRODUCTION OR USE OF THE INFORMATION AND DATA CONTAINED HEREIN IS NOT PERMITTED FOR ANY PURPOSE OTHER THAN THAT PROPOSED BY THE STUDY WITHOUT THE PRIOR FORMAL AUTHORIZATION OF THE AUTHORS .

.0 abrilversion of2 of 2019

STUDY FLOW CHART

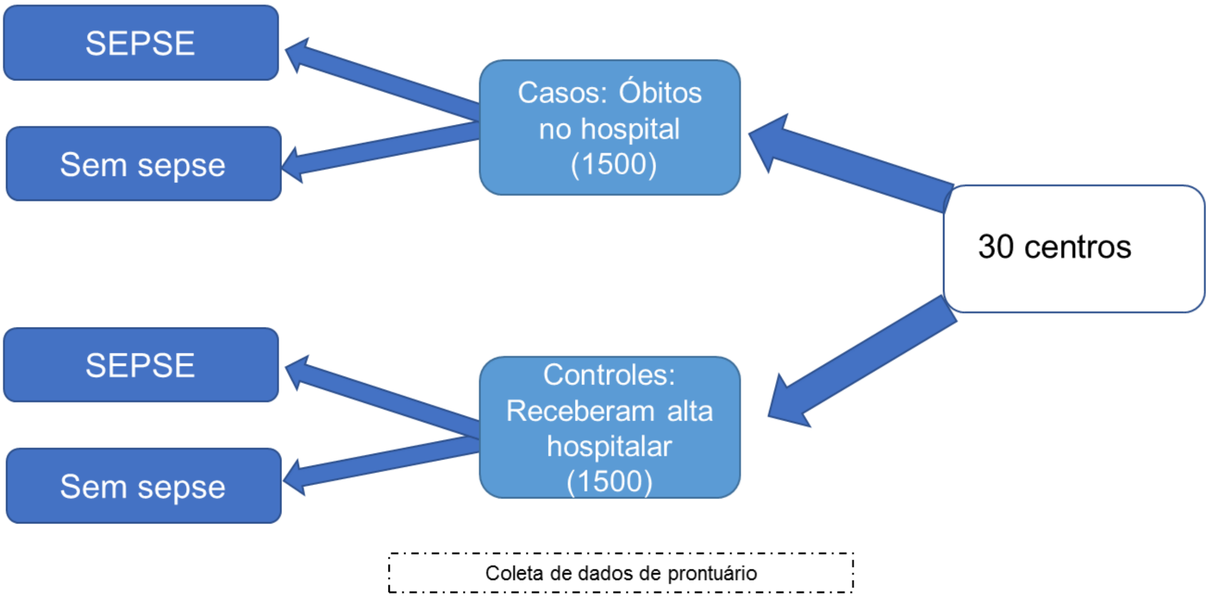

**Title**	**IMPACT-MAPA Study**<br>**Identifying Sepsis -Attributable Mortality in Hospitalized Patients in Brazil**<br>**A study of the Platform of Support Projects for the National Action Plan for the Prevention and Control of Antimicrobial Resistance – IMPACTO MR Program**
**Coordinating Center**	Heart Hospital Research Institute (IP-HCor)<br>Rua Abilio Soares, 250 – Paraíso<br>CEP: 04005-000 São Paulo, SP – Brazil<br>Tel : 11 3053 6611 Extension: 8210<br>Fax: 11 3886 4695
**Study Design**	Observational case-control study
**Primary Purpose**	To measure attributable mortality from nosocomial sepsis in hospitalized patients in Brazil.
**secondary objectives**	1. Assess length of hospital stay in patients with or without sepsis<br>2. Assess mortality attributable to other hospitalization complications (infarction, stroke, pulmonary thromboembolism)<br>3. Evaluate the quality of completion of medical records<br>4. Assess the quality and consistency of filling in the hospital death certificate
**Eligibility of cases and controls**	Each hospital will provide clinical, demographic and laboratory data for the last 50 deaths that occurred in the institution. Data from the patient who was discharged alive with a date and time closer to the date of death of the case will be used as controls.
**sample size**	30 hospitals, 100 patients in each hospital (50 cases and 50 controls), totaling 3,000 patients

SUMMARY

INTRODUCTION........5
STUDY OBJECTIVES........6
METHODS........7
STUDY DESIGN........7
SAMPLE SIZE........7
DATA COLLECTION........9
STATISTICAL ANALYSIS........12
DATA MANAGEMENT........13
STUDY SCHEDULE........15
DISSEMINATION OF RESULTS........16
PROJECT RISKS........16
ETHICAL CONSIDERATIONS........17
REFERENCES........18

Introduction

Hospital admission is not without risks. Admitted patients are subject to a myriad of clinical complications that can represent important health problems, leading to an increase in morbidity and mortality. These complications are also associated with increased costs, caused not only by the increase in hospitalization time but also by the need for additional procedures and treatments, in addition to the greater absence of the patient from his usual activity. Thus, it becomes important to understand the frequency and contribution of clinical complications in hospitalized patients.

Among the most frequent complications, the occurrence of nosocomial infection stands out. In developing countries, nosocomial infections are extremely frequent, reaching more than 15 in every 100 hospitalized patients [1]. In some more severe situations, the infectious condition generates dysfunction of one or more organs, a situation called sepsis and which has high morbidity and mortality [2]. Despite its frequency, the attributable mortality of sepsis, that is, the increase in mortality that results from the occurrence of nosocomial sepsis in a patient, is unknown.

We have a lot of information on the prevalence and incidence of sepsis, and lethality in patients with sepsis, including good data from Brazil [3]. We also have some data on mortality attributable to sepsis among patients with sepsis, that is, of the total number of deaths that can be directly attribute to sepsis rather than other concomitant problems. For example, in patients

admitted to the ICU, the mortality fraction attributable to sepsis is approximately 15% in developed countries [4]. However, from the point of view of health management, it is essential to understand what proportion of the total number of deaths in the population can be attributed to sepsis. This information is obviously critical for decisions about allocating investments in assistance, training and research. However, there is no good quality information on the proportion of deaths that are attributable to sepsis, not even from other countries. Our proposal is to estimate, among the cases of hospital deaths, what proportion is attributable to sepsis versus other groups of causes (cardiovascular diseases, cancer, etc.).

Understanding attributable mortality from sepsis has several relevant operational and academic implications. From an operational point of view, the measurement of the attributable mortality fraction allows estimating the impact of future interventions aimed at reducing the occurrence of sepsis (handwashing campaign, actions by the Hospital Infection Commission, among others). From an academic point of view, this information will allow a better sample size calculation for future clinical trials in the area.

Study objectives

Primary: To measure attributable mortality from nosocomial sepsis in hospitalized patients in Brazil.

Secondary:

1. Length of hospital stay in patients with or without sepsis
2. To assess mortality attributable to other hospitalization complications (infarction, stroke, pulmonary thromboembolism)
3. Evaluate the quality of completion of medical records
4. Assess the quality and consistency of filling in the hospital death certificate

Methods

Study design

Case-control study to be carried out through analysis of medical records.

Sample size

The sample size calculation was performed using the Fleiss , Tytun and Ury method to estimate the power of a two-tailed test for proportion difference using the Hmisc package of the R software (version 3.5.0). The sample size was estimated for a power above 90% to detect an *odds ratio* of at least 1.4 assuming an incidence of nosocomial sepsis in controls of 15%. Two thousand seven hundred patients would be needed. We have chosen to round the number to 3,000 in order to ensure a safety margin.

Eligibility criteria for institutions

This total of patients will be included in a group of 30 Brazilian hospitals that meet the following characteristics:

1. At least 100 active beds
2. Presence of intensive care unit and /ouemergency room
3. Availability to collect data for the study

Eligibility criteria for patients

Inclusion criteria

Each hospital will provide clinical, demographic and laboratory data for the last 50 deaths that occurred in the institution. Data from the patient who was discharged alive with a date and time closer to the date of death of the case will be used as controls. Fifty controls will be used per hospital, totaling 100 patients per hospital. Consecutive matching with the closest live high will ensure that any temporal impacts are minimized.

In addition to the general criteria mentioned, the specific inclusion criteria are:

- Patients over 18 years of age;
- Hospital stay for more than 24 hours;

Exclusion criteria

Discharge cases due to transfer, evasion or home care should be excluded..

Data collection

Data collection will be performed by each hospital using an electronic data collection system designed specifically for the study. The filling will be done by each center based on data from medical records, according to local availability, respecting the maximum period of completion of two years. All data collected will be anonymous, in order to preserve the identity of the participants and guarantee confidentiality. The data to be collected are:

1. Charlson Comorbidity Index)
2. Reason for admission: Scheduled versus unscheduled. Presence of infection as the cause of hospital admission (defined as admission with a primary diagnosis of infectious pathology by ICD 10).
3. Physiological variables: Blood pressure, heart rate, respiratory rate, level of consciousness.
4. Laboratory variables: Renal function (serum creatinine), blood count, C-reactive protein (if available), coagulogram , bilirubin, serum lactate .
5. Result of all cultures (blood, tracheal secretion, urine or other sites) collected during hospitalization.
6. Length of stay in hospital
7. Use of intra-hospital resources

a. Number of imaging tests performed to elucidate an infectious focus

b. Need for surgical procedures

c. Intensive care stay

d. Use of supportive therapy such as mechanical ventilation, renal replacement therapy, use of vasopressors.

Laboratory and physiological variables will be collected from admission and during the first three days of the patient's hospitalization. In the event of addition, initiation or change of antimicrobial regimen and/or collection of some type of microbiological examination (culture), we will perform physiological and laboratory data collection again for an additional period of three days before and after the date of your occurrence. in order to monitor the occurrence of organic dysfunction associated with a suspected infectious condition (sepsis). In these situations, we will also note:

1. The causative focus of sepsis
2. The antibiotic prescribed and its suitability (i.e., whether the antibiotic prescribed at the initial time was effective against the pathogen that eventually proved to be the cause of the event)
3. Whether or not the infection was associated with a surgical procedure or invasive device (prosthesis infection, catheter-related bloodstream infection, etc.).

We will also note the occurrence of other clinical complications, such as:

1. Pulmonary thromboembolism
2. Myocardial ischemia/myocardial infarction
3. Stroke (ischemic or hemorrhagic)

For cases (that is, for hospital deaths), we will collect the information contained in the death certificate (immediate cause and its sequence, as well as contributing causes).

Definition of the exposure factor: The exposure of interest is nosocomial sepsis, that is, a nosocomial infection that courses with the occurrence of organic dysfunction. As organic dysfunction, we will consider the presence of at least one of the factors below when starting or changing the therapeutic antimicrobial regimen in a hospitalized patient:

1. Systolic pressure < 90 mmHg or mean pressure < 65 mmHg or pressure drop > 40 mmHg
2. PaO2 /FiO2 ratio <300 or O2 requirement to maintain SpO2 > 90
3. Lowering the level of consciousness
4. Creatinine > 2.0 mg /dL or urine output less than 0.5mL/Kg/h in the last 2 hours
5. Bilirubin > 2mg/dL
6. Platelet count < 100,000mm3

7. Lactate above the reference value
8. Coagulopathy (INR > 1.5 or APTT > 60 sec)

Statistical analysis

Traditional univariate analysis comparing groups (case and control) with respect to the variables described above will be performed using t-test or Mann-Whitney test for continuous variables (as appropriate depending on the occurrence of normality) and Chi-square test for comparison of proportions.

The following approach will be used to calculate attributable fraction calculation for each admission type: The inverted probability weights (IPW) for each patient will be calculated, representing the cumulative risk of the patient acquiring sepsis during hospitalization, under a multivariable logistic regression analysis including baseline age, Charlson Comorbidity Index, and a time-dependent variable for the occurrence of clinically relevant events. We will estimate the association between sepsis occurrence on hospital mortality within 28 days through a mixed logistic regression model weighted by IPW. The model will include the participant center as a random intercept, and age, Charlson Comorbidity Index, infection at admission, IPW, and the accumulated dependent time variables of sepsis occurrence and clinically relevant events with their interaction with time (modeled with a third-degree polynomial). From the daily odds ratios

estimated by the model, the Miettinen formula will be used to calculate the attributable mortality of sepsis:

$$P_{c_i} * \frac{OR_i - 1}{OR_i}$$

$$i = day,\ P_c = proportion\ of\ patients\ that\ had\ sepsis\ among\ non - survivors$$

We will also present the marginal effect of having one sepsis episode on the whole period, with an estimate of average attributable fraction obtained from the model. All analyses will be performed using the R software

Sensitivity analysis: Different definitions of sepsis will be explored; the same analysis method described above will be used for different definitions as sensitivity analyses. An internal consensus was created within the steering committee on probability degrees of sepsis according to combinations of use of antibiotics, organ failure, results of cultures, and occurrence of clinically relevant events. Two sensitivity analyses will be made, one estimating the attributable fraction considering definitive, very probable, and probable sepsis, and a second sensitivity analysis considering only definitive and very probable sepsis.

Data management

All data will be included in an electronic system available in the cloud developed specifically for the study. Measures to ensure the completion and quality of data will be employed, including:

1. Face-to-face and/or distance training for all investigators
2. Conducting a meeting of researchers
3. Remote data completion monitoring
4. Frequent contact with centers (face-to-face and remotely)
5. Audit and adjudication of exposure of interest (sepsis) in all cases.

Study Schedule

The total duration of the study is 03 years, with the following schedule:

Year/Semester	Year 1/1	Year 1/2	Year 2/1	Year 2/2	Year 3/1	Year 3/2
**project coordination**						
project management	X	X	X	X	X	X
Quarterly meetings with MS technicians	X	X	X	X	X	X
Annual meetings with the Steering Committee	X	X	X	X	X	X
**Approval of centers**						
Initial protocol submission at the coordinating center	X	X	X	X	X	X
Monitoring of protocol submissions and approvals at the centers	X	X	X	X	X	X
Submission of partial and final reports to the CEP	X	X	X	X	X	X
**Center management (patient inclusion)**						
Selection of centers	X	X				
Graphic design and printing of support material	X					
Center training	X	X	X	X	X	X
Stimulate patient inclusion	X	X	X	X	X	X
Assistance to researchers in conducting the protocol	X	X	X	X	X	X
monitoring	X	X	X	X	X	X
Payment to investigators	X	X	X	X	X	X
**Data Management and Results Analysis**						
CRF preparation (printed and electronic)	X	X				X
System maintenance	X	X	X	X	X	X
data cleaning		X	X	X	X	X
Database closure					X	X
Data analysis						X
**Publication**						
Submission of results for publication/congress						X

Dissemination of Results

The Steering Committee of the IMPACTO MR-MAPA Study is committed to publishing its results, whatever they may be. As this is a large-scale, collaborative, randomized study, we aim to refer the main publications to high-impact journals. Study data will be immediately made available to the Ministry of Health.

We intend to open access to the study database to other researchers. In the first two years after the publication of the main manuscript, the investigators will dedicate themselves to carrying out analyzes for substudies proposed by investigators of the collaborative group. At this stage, the database will be kept under the custody of the study coordinators, and its access will be allowed to third parties only with the express authorization of the Steering Committee of the IMPACTO MR-MAPA study after evaluation of the proposal accompanied by a statistical analysis plan.

Project Risks

We believe that the IMPACTO MR-MAPA study presents a low risk. We will perform a retrospective analysis using medical records in 30 Brazilian hospitals. Data collection will be done anonymously through a computerized central system. All analyses will be performed without exposing the patients' names or individualized information of the participating hospitals. The data collection system will be designed in a way that minimizes the input of

incorrect information. The data inserted will be checked by the data management of the study.

Ethical considerations

This is a case-control study, through a retrospective analysis of medical record data, with anonymous data collection. Thus, we anticipate that the risk to patients and institutions is minimal. Due to the very nature of the study, we will request exemption from obtaining the free and informed consent form from the centers. There will be no contact or interaction with the patients included.

References

1. Allegranzi B, Bagheri Nejad S, Combescure C, Graafmans W, Attar H, Donaldson L, Pittet D. Burden of endemic health-care-associated infection in developing countries: systematic review and meta-analysis. Lancet. 2011 Jan 15 ;377 (9761):228-41

2. Singer M, Deutschman CS, Seymour CW, Shankar- Hari M, Annane D, Bauer M, Bellomo R, Bernard GR, Chiche JD, Coopersmith CM, Hotchkiss RS, Levy MM, Marshall JC, Martin GS, Opal SM, Rubenfeld GD, van der Poll T, Vincent JL, Angus DC. The Third International Consensus Definitions for Sepsis and Septic Shock (Sepsis-3). JAMA. 2016 Feb 23;315(8):801-10

3. Machado FR, Cavalcanti AB, Bozza FA, Ferreira EM, Angotti Carrara FS, Sousa JL, Caixeta N, Salomao R, Angus DC, Pontes Azevedo LC; SPREAD Investigators ; Latin American sepsis Institute Network. The epidemiology of sepsis in Brazilian intensive care units (the Sepsis PREvalence Assessment Database, SPREAD): an observational study. Lancet Infect Dis. 2017 ;17 (11):1180-1189

4. Shankar-Hari M, Harrison DA, Rowan KM, Rubenfeld GD. Estimating attributable fraction of mortality from sepsis to inform clinical trials. J Crit Care. 2018 Jan 25 ;45:33 -39
